# TraVis Pies: A Guide for Stable Isotope Metabolomics Interpretation Using an Intuitive Visualization

**DOI:** 10.3390/metabo12070593

**Published:** 2022-06-25

**Authors:** Sam De Craemer, Karen Driesen, Bart Ghesquière

**Affiliations:** 1 Metabolomics Expertise Center, VIB Center for Cancer Biology, 3000 Leuven, Belgium; karen.driesen@kuleuven.be; 2 Metabolomics Expertise Center, Department of Oncology, Katholieke Universiteit Leuven, 3000 Leuven, Belgium

**Keywords:** tracer metabolomics, data visualization, biochemical pathways

## Abstract

Tracer metabolomics is a powerful technology for the biomedical community to study and understand disease-inflicted metabolic mechanisms. However, the interpretation of tracer metabolomics results is highly technical, as the metabolites’ abundances, tracer incorporation and positions on the metabolic map all must be jointly interpreted. The field is currently lacking a structured approach to help less experienced researchers start the interpretation of tracer metabolomics datasets. We propose an approach using an intuitive visualization concept aided by a novel open-source tool, and provide guidelines on how researchers can apply the approach and the visualization tool to their own datasets. Using a showcase experiment, we demonstrate that the visualization approach leads to an intuitive interpretation that can ease researchers into understanding their tracer metabolomics data.

## 1. Introduction

Metabolomics is key to understanding the biochemical changes that contribute to disease [1]. Consequently, there is a great deal of interest from the medical community in the available techniques that identify these metabolic alterations. In particular, the technology of tracer metabolomics is of great interest as it provides the ability to not only identify the relative differences in abundances of metabolites between healthy and diseased systems, but it can also reveal insights into the connectivity of pathways. The latter is essential to understand how the complex intertwined network of biochemical reactions is contributing to the phenotypic changes [2]. At the core of this technology lies the administration of an isotopically non-radioactive labeled nutrient (tracer), most commonly ^13^C, to biological systems, such as cell cultures or organisms. For each metabolite that is biochemically linked to this nutrient, we investigated all of the potential combinations of the numbers of light and heavy isotopes for this metabolite, known as the isotopologues. For example, a compound with three carbons has four carbon isotopologues, respectively, with 0, 1, 2 or 3 out of the 3 carbons labeled and these can be discerned by mass spectrometric (MS) techniques. Eventually, we obtained the abundance of the metabolite (relative or absolute) and the labeling patterns, i.e., the contribution of each isotopologue present to the total abundance. The alterations were identified by combining these two levels of information with the known or assumed position of the metabolites in the biochemical network.

However, the holistic interpretation of these three levels of information is complex. This proves to be a barrier to harvesting fully the results that the field of tracer metabolomics produces, by researchers who are usually experts on the systems they study rather than on the analytical techniques used to study them. Both the interpretation and communication of these results would benefit from an intuitive visualization, and from a tool to generate this. This is of special interest for the communication between the analytical experts or researchers who obtain compound abundances and labeling patterns from the raw measurement data, and the biomedical researchers who need to interpret these in their biological context. This is even more true when dealing with non-scientific stakeholders or the general public.

The publicly available application Escher-Trace is an excellent tool to visually compare the labeling or abundances of metabolites between different systems (cohorts) on metabolic maps, among other functionalities [3]. However, the output still requires the viewer to crosscheck graphs suited for labeling information with graphs suited for metabolite abundance. This is confusing for researchers who are new to the field or who only rarely encounter it, and who would benefit from a visualization that conveys both the abundance and labelling information at a glance, while intuitively showing the relation between the two. Such a visualization would help them to understand their data and prepare them for tackling the more detailed interpretation of their results.

A first step towards such a visual is to simplify the labeling pattern to a metric called the fractional contribution. This was described for carbon by Buescher et al. (2015) [2] as the fraction of a metabolite’s carbon coming from a certain nutrient; in practice, the ^13^C carbon coming from the labeled nutrient, and is calculated according to the formula below:

(1) $$FC=\frac{\sum_{i=0}^{n}i*m_{i}}{n*\sum_{i=0}^{n}m_{i}}$$

With *FC* being the fractional contribution; *n* the number of C atoms in the metabolite; *i* denoting the isotopologues; and *m_i_* the abundance of isotopologue *i*. An example of a graph combining abundance and fractional contribution is a pie chart of which the radius corresponds to the abundance, while the size of the slices corresponds to the fractional contribution. Similar visualizations have previously been used to present tracer metabolomics’ results, although rarely and without statistically significant information [4,5]. The lack of a freely available tool, and the fact that generating these graphs manually is extremely tedious, limit their application.

Here, we provide a tutorial for using this type of visualization to interpret tracer metabolomics’ data. We provide a rationale for the visualization chosen, then introduce TraVis Pies, a tool to generate these visualizations from curated tracer metabolomics’ data. We then demonstrate how to pinpoint quickly metabolic changes using the visualization by TraVis Pies, by applying it to an in vitro experiment comparing murine 4T1 breast cancer cells treated with well-known inhibitors versus untreated cells. We then showcase an example of how to proceed once the metabolites or pathways of interest have been identified, by moving beyond the fractional contribution and looking into the tracer labelling patterns. To aid researchers in applying this methodology to their own data, we provide a guide to the interpretation and the visualization tool in the Supplementary Materials supplements for this article.

## 2. Results and Discussion

### 2.1. Visualization Rationale

To allow less-experienced researchers to identify quickly the metabolites or pathways of interest, and to facilitate communication with people unfamiliar with tracer metabolomics, there is a need for a visualization that intuitively conveys what the values coming out of a tracer metabolomics’ experiment mean. This presents three criteria for the visualization: (1) the relation between the abundance and fractional contribution of a compound should be visually immediately apparent; (2) the differences in both the abundance and fractional contribution between different cohorts should be visually immediately apparent; and (3) it should be easy to compare these metrics and their patterns over different cohorts between multiple metabolites. In addition, there should be a way to verify if the visually apparent difference is statistically significant. Here, we discuss some graphs that we considered, of which a visual representation on the glycolysis pathway is given in the Supplementary Materials, Section 4.

The XY scatterplots, with one axis presenting the abundance and the other presenting the fractional contribution, would be the most concise way of presenting the different metrics for different groups in one plot. They can also easily incorporate confidence intervals or significance *p* values. However, they do not conform to criterion (1), as it is not visually apparent that the fractional contribution is a fraction of the abundance. This adds a level of abstraction to the visualization that we wish to avoid. Criterion (3) is also not fully satisfied, as by nature the position of the point representing a certain cohort in the visual will change depending on the metrics, which makes it harder to follow quickly the trends for specific cohorts over different metabolites.

Other options are: (A) A stacked bar plot with one bar per cohort, where the bar width corresponds to the abundance and the total bar height is always one with individual bar heights corresponds to the size of the labeled and unlabeled fraction; and (B) One pie chart per cohort with the radius corresponding to the relative abundance, and with slices corresponding in size to the labeled and unlabeled fractions. Both representations conform to all three of the criteria: the fractional contribution is shown clearly as a fraction of the total abundance; it is easy to compare both of the metrics between different cohorts, and each cohort has a fixed place in the representation. While the confidence intervals cannot be visualized neatly on these types of graphs, the *p* values can be printed on them, since a lot of space is allocated to each cohort. The bar plot presentation with changing bar widths is uncommon while most people are familiar with pie charts, that are already being used in the tracer metabolomics field [4,5], so we decided to work with the latter.

### 2.2. Travis Pies, a Software to Generate the Proposed Visualizations

When trying to generate these visualizations, we noticed a lack of software that would allow the production of this type of plot automatically from curated tracer metabolomics datasets based on abundance, fractional contribution data and metadata. Therefore we developed TraVis Pies, an interfaced visualization tool written in R that generates a set of a pie charts from curated tracer metabolomics’ data, that can be applied to any tracer experiment, regardless of which labeled element and which nutrient is used.

The tool is open source and freely available, see the Software Availability Statement. More information on how TraVis Pies operates can be found in Section 3.4 of this manuscript, or in the most detail in Section 3 of the Supplementary Materials.

### 2.3. Visualization Application and Interpretation

**Figure 1:**
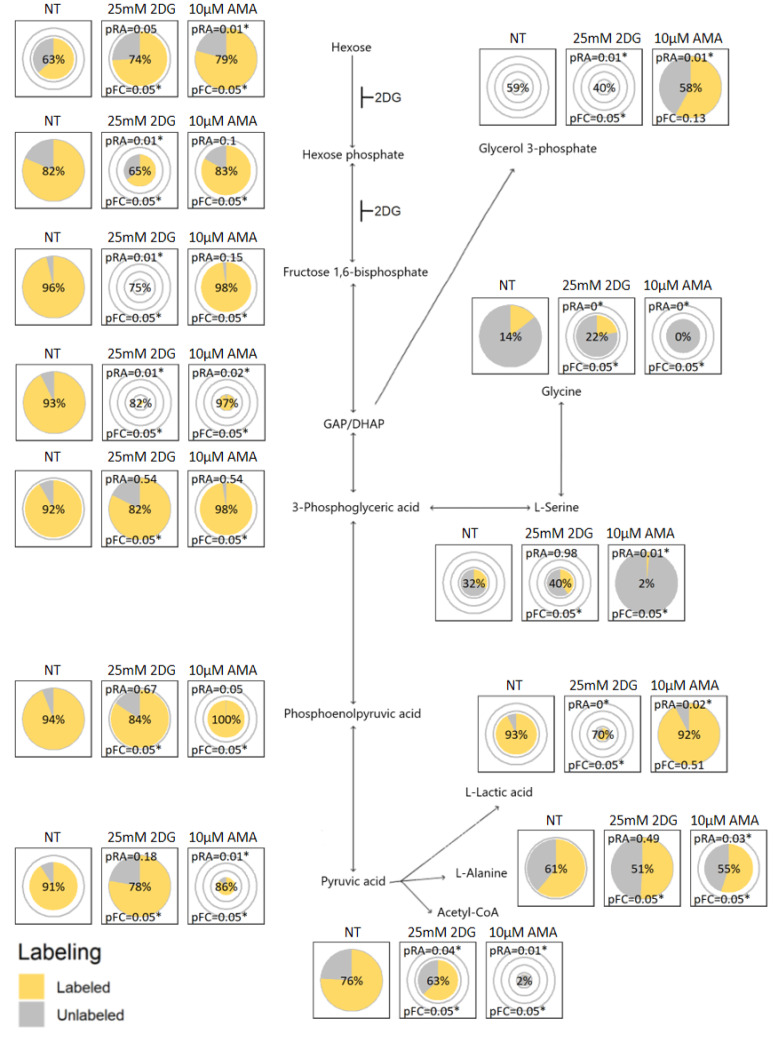
Visualizing tracer incorporation in glycolysis: Proposed pie chart visualizations by TraVis Pies plotted on a metabolic map of the metabolization of ^13^C_6_-labelled glucose into glycolysis applied to three cohorts: non-treated (NT); 25 mM 2-deoxyglucose treated (2DG); or 10 µM antimycin A treated (AMA). For each metabolite, the pie radii correspond to the relative abundance which can be compared between the cohorts of this metabolite. Both the labeled surface fraction of the pie (yellow) and the percentage displayed in the middle of each pie reflect the fractional contribution. pRA and pFC indicate the significance of the difference in, respectively, the relative abundance or fractional contribution with the NT cohort. * indicates a *p* value ≤ 0.05. The details of the visualization are explained in more detail in the Methods section. GAP/DHAP: figure for either D-Glyceraldehyde 3-phosphate or Dihydroxyacetone phosphate, that are in equilibrium.

**Figure 2:**
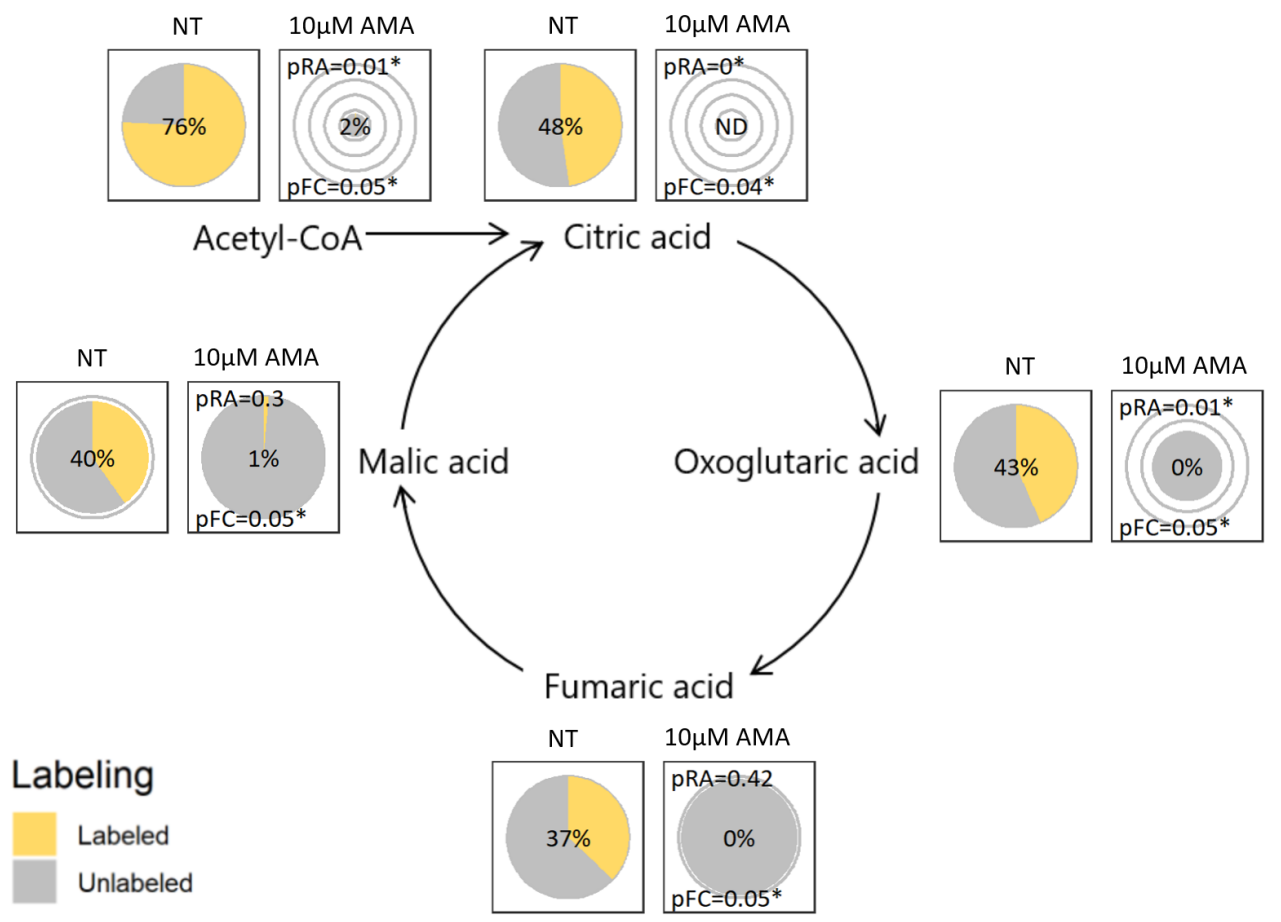
Visualizing tracer incorporation in the TCA cycle: Proposed pie chart visualization plotted on a metabolic map of the metabolization of Acetyl-CoA applied to two cohorts: non-treated (NT) or 10 µM antimycin A treated (AMA). Pie diameter shows the relative abundance of this compound, while the size of the yellow slice of the pie relative to the total size and the label in the center show the fractional contribution. pRA and pFC indicate the significance of the difference in, respectively, the relative abundance or fractional contribution with the NT cohort. * indicates a *p* value ≤ 0.05. The details of the visualization are explained in more detail in the Methods section. ND: not detected in any sample of this cohort.

Figure 1 and Figure 2 illustrate the use of the visualization concept on two different pathways by comparing ^13^C_6_-glucose metabolization in murine 4T1 breast cancer cells that were either: non-treated (NT); 25 mM 2-deoxyglucose treated (2DG); or 10 µM antimycin A treated (AMA). The 2DG is expected to inhibit the conversion of glucose to glucose 6-phosphate, and of the latter to fructose 6-phosphate [6], hence blocking glycolysis. The AMA is expected to inhibit complex III of the respiratory chain, reducing the regeneration of NAD+ from NADH, which is required to maintain glycolysis and consequently inhibiting the TCA cycle [7]. The pie charts positioned on the metabolic maps in Figure 1 and Figure 2 were produced per metabolite using TraVis Pies. In the Supplementary Materials, Section 2.2.2, we provide a detailed tutorial on how the conclusions regarding the aforementioned experiments have been obtained from these figures. This will aid researchers in the correct interpretation of their own tracer experiments.

**Figure 3:**
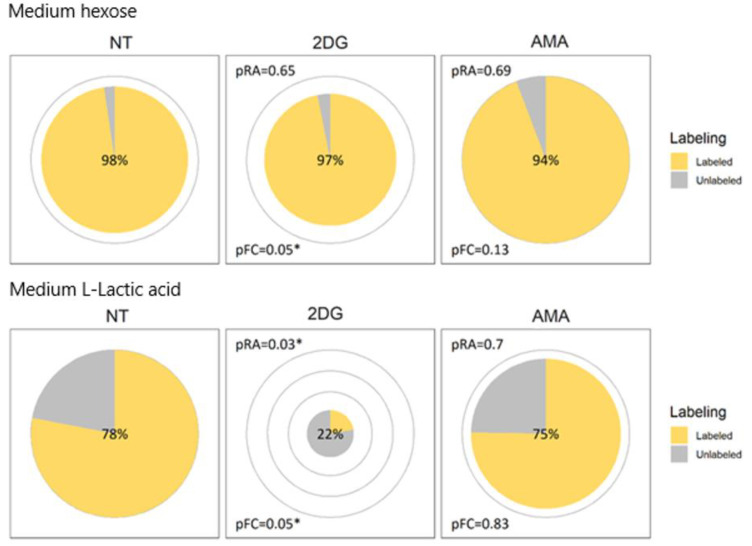
Proposed pie chart visualization for medium hexose and lactic acid applied to three cohorts: non-treated (NT); 25 mM 2-deoxyglucose treated (2DG); or 10 µM antimycin A treated (AMA). For each metabolite, the pie radii correspond to the relative abundance which can be compared between cohorts of this metabolite. Both the labeled surface fraction of the pie (yellow) and the percentage displayed in the middle of each pie reflect the fractional contribution. pRA and pFC indicate the significance of the difference in, respectively, the relative abundance or fractional contribution with the NT cohort. * indicates a *p* value ≤ 0.05.

Figure 1 shows the effect of 2DG and AMA on the glycolytic pathway by combining the relative changes in abundance with the fractional labeling. As expected, the 2DG blocks glycolysis, which is evidenced by a drop in the abundance of the metabolites, hexose phosphate, hexose-1,6-bisphosphate and GAP/DHAP, as well as a reduction in the secretion of extracellular lactic acid (Figure 3). Interestingly, following the aldolase step, that catalyzes the conversion of fructose 1,6-bisphosphate into the GAP/DHAP, a recovery is observed in the abundances of the ‘downstream’ metabolites 3-phosphoglyceric-, phosphoenolpyruvic- and pyruvic acid.

The metabolic effect of AMA on the TCA cycle is shown in Figure 2. The treatment seems to affect the entry of carbons from glucose into the TCA cycle through the inhibition of pyruvate dehydrogenase, and consequently results in a significant decrease in the citric acid levels. Yet, a compensation of the AMA inhibition seems to occur at the level of oxoglutaric acid, which can most likely be explained by glutamine anaplerosis of the TCA cycle, a pathway shuttling unlabeled carbons from glutamine (^12^C) into the pool of oxoglutaric acid. The effect of AMA goes beyond the TCA cycle, as shown in Figure 1, where the treatment also results in a drop in the pyruvic acid and an increase in the intracellular lactic acid, as well as a significant increase in the abundance of glycerol-3-phosphate. The latter can be indicative of an activation of the glycerol 3-phosphate shunt that becomes activated restoring the intracellular NAD:NADH ratio.

In the NT conditions, hexose phosphate seems to have more labeling than hexose. The reason is probably that it is not only glucose (phosphate) that contributes to the hexose (phosphate signals), but also the other hexose (phosphates), that are less likely to have labeled carbons incorporated. The employed LC–MS method is not specifically tailored for separating the isomeric species of hexoses or hexose phosphates from each other, in the same way as most methods that try to cover a broad range of metabolites. Medium hexose is similarly abundant and almost fully labeled in all of the conditions (Figure 3), as the labeled glucose that is added to the medium dominates the other hexoses. The downstream metabolites, such as fructose 1,6-bisphosphate and DHAP, are almost fully labeled in the healthy cell extracts as expected, demonstrating that the glucose (phosphate) fractions of the hexose (phosphates) were almost fully labeled as well.

Note that the fractional contribution reflects the amount of carbons coming from a labeled nutrient only when that nutrient is fully labeled (e.g., ^13^C_6_-glucose or ^13^C_5_-glutamine). When using partially labeled tracers (e.g., 1-^13^C glucose) this is no longer the case, and the usefulness of the fractional contribution diminishes. This is not really an issue as the partially labeled tracers are typically used in experiments that follow up on a full tracer experiment and further test the obtained hypotheses from the fully labeled nutrients. Indeed, these partially labeled tracers generate discriminating isotopologues of metabolites that would only be produced through the hypothesized pathway, and not by potential alternative metabolic routes [8,9,10].

### 2.4. Verification by Labeling Patterns

At this point, further verification is still needed by looking into the labeling patterns themselves, as they might provide additional insights into the pathway activities and pick up relevant biochemical differences that are not revealed by the fractional contribution. For this reason, the labeling patterns should be checked for all of the compounds in the study, although one would start with those compounds that seemed interesting based on the previous step. One built-in aid in the tool is the option to add * to the names of cohorts that have at least one isotopologue fraction significantly different (*p* < 0.05) from the reference cohort.

In the described experiment, most of the compounds are either fully labeled or fully unlabeled. For these compounds, the information from the labeling pattern is that either all of the carbons are coming from the nutrient, or all of them are coming from a different carbon source.

**Figure 4:**
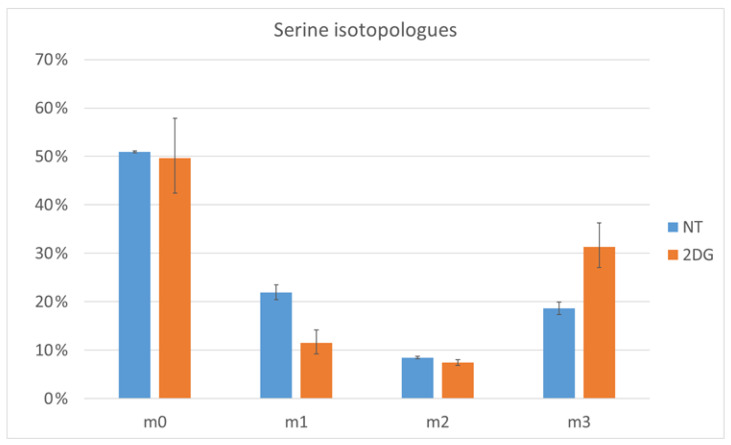
Serine-labeling pattern in non-treated (NT) and 2DG treated cells (2DG). The error bars mark the 95% confidence intervals.

An example where the labelling pattern provides additional insights is the amino acid, serine. The labeling pattern is shown in Figure 4. While the fractional contribution is higher for 2DG, looking into the labeling pattern reveals that there is little or no difference in the fraction of serine that is fully unlabeled. In addition, isotopologues with one or two labeled carbons are also present. While 2DG treated cells have more serine, with three labeled carbons, than the NT cells, the latter have more serine with one labeled carbon (m1 isotopologue). A candidate pathway for the generation of the m1 isotopologue is the non-phosphorylated synthesis of serine from unlabeled glycine and ^13^C-N^5^N^10^-methylene tetrahydrofolate.

### 2.5. Support for Application on Other Experiments

As demonstrated, the integrated visualization facilitates the interpretation and communication of tracer metabolomics results in a biomedical context, and provides a tool for biomedical researchers and analytical experts or researchers to interact constructively to dissect the biochemical mechanisms. In order to support the use of this visualization approach, we provide: (1) TraVis Pies as an open source, interfaced R script with a readme file to aid installation and usage (at the time of writing we are also preparing to launch it as a web tool, see Software Availability Statement); (2) a theoretical and applied guide on the interpretation of plots generated by TraVis Pies in the Supplementary Materials, Section 2.

## 3. Materials and Methods

### 3.1. Cell Culture Experiments

The murine 4T1 cells were purchased from ATCC (American Type Culture Collection, Manassas, VA, USA). The cells were cultured for 24 h in the presence of 25 mM fully labeled ^13^C_6_-glucose dissolved in DMEM (DMEM no glucose, ThermoFisher Scientific, Waltham, MA, USA), supplemented with 10% fetal bovine serum in the specified conditions (see further). All of the setups were run in triplicate, with an additional setup only containing ^12^C_6_- glucose as a control sample for potential interferences during LC-MS analysis. For each setup, 500 k cells were plated in a 6-well cell-culture plate, to which the following conditions were applied: non-treated (NT); 25 mM 2-deoxyglucose (Sigma-Aldrich, St. Louis, MO, USA); or 10 µM Antimycin A (Sigma Aldrich).

Following treatment, the medium was removed and the cells were washed with ice-cold NaCl (150 mM in water). Following removal of the washing buffer, extraction was completed in 300 μL of an 80% methanol (LC–MS grade methanol, VWR, Leuven, Belgium) extraction buffer containing internal standards (3 µM ^13^C_5_-D_5_-^15^N Glutamic acid, 3 µM D_7_-^15^N_4_ Arginine and 5 µM D_7_-Glucose). The extracts were placed in −80 °C overnight to ensure full protein precipitation. After, the samples were centrifuged (15,000 rpm, 15 min, 4 °C) using a tabletop centrifuge and the supernatant was transferred to appropriate MS vials.

A total of 200 μL of 200 mM NaOH solution was added to the protein pellets. The pellets were then heated for 30 min at 95 °C to solubilize the proteins and subsequently cooled down on ice. Then, the pellets were centrifuged again (5000 rpm, 10 min, 4 °C). The supernatant was used to determine protein concentration (BCA assay, Pierce, Appleton, WI, USA), which was used for normalization purposes by the TraVis Pies tool.

### 3.2. LC-MS Method

A total of 10 µL of each sample was loaded into a Dionex UltiMate 3000 LC System (Thermo Scientific, Bremen, Germany) equipped with a C-18 column (Acquity UPLC -HSS T3 1. 8 µm; 2.1 × 150 mm, Waters, Milford, MA, USA) coupled to a Q Exactive Orbitrap mass spectrometer (Thermo Scientific), operating in negative ion mode. A step gradient was carried out using solvent A (10 mM TBA and 15 mM acetic acid) and solvent B (100% methanol). The gradient started with 5% of solvent B and 95% solvent A and remained at 5% B until 2 min post injection. A linear gradient to 37% B was carried out until 7 min and increased to 41% until 14 min. Between 14 and 26 min the gradient increased to 95% of B and remained at 95% B for 4 min. At 30 min the gradient returned to 5% B. The chromatography was stopped at 40 min. The flow was kept constant at 0.25 mL/min and the column was placed at 40 °C throughout the analysis. The MS operated in full scan mode (m/z range: (70.0000–1050.0000)) using a spray voltage of 4.80 kV, capillary temperature of 300 °C, sheath gas at 40.0, auxiliary gas at 10.0. The AGC target was set at 3.0 × 10^6^ using a resolution of 140,000, with a maximum IT fill time of 512 ms.

### 3.3. Data Acquisition and Preliminary Analysis

The data collection during the LC–MS measurement was performed using the Xcalibur software (version 4.2.47, Thermo Scientific). Raw MS tracer data were processed to mzmL files, using Proteowizard’s MSConvert tool. The peak picking was completed using these files in El-Maven (Elucidata, Cambridge, MA, USA). The calculation of abundances, fractional contributions and labeling profiles from the El-Maven output was completed in the Polly Labeled LC-MS Workflow (Elucidata). Internal standards signals were reproducible over the samples, so no normalization was performed on them.

### 3.4. Calculating the Fractional Contribution of Acetyl-CoA

The acetyl-Coenzyme A (acetyl-CoA) is formed when Coenzyme A (CoA) binds with an acetyl group coming from a substrate (pyruvic acid, fatty acids, citric acid). While studying the aforementioned pathways, the main interest was the acetyl group, especially when using ^13^C glucose. In tracer metabolomics, we would be interested in knowing to what extent the acetyl group gets labeled. However, acetyl-CoA contains other carbons that can be derived from the same nutrients that label the acetyl group. For example, the phosphoadenosine moiety of CoA can incorporate carbons coming from glucose. It is thus very hard to obtain a labeling pattern or fractional contribution for the acetyl carbons separately, especially because Formula (1) is taking all of the carbons into account, and not the quantity of carbons from a molecule that can potentially carry the ^13^C atoms with the given tracer.

In order to tackle this issue, there are examples in the literature that correct for this by looking at the labeling patterns in related metabolites [11]. We opted for a simpler approach by comparing the acetyl-CoA with CoA labeling patterns, such as the reference above, assuming that the acetyl is coming from pyruvate and is either fully labeled (^13^C_2_), or fully unlabeled (^12^C_2_), and no partially labeled form (^13^C^12^C) is present. If we note the fraction of acetyl-CoA (AC) that has *n* labeled isotopes $F_{AC}^{n}$, and use similar notations for the acetyl (A) and CoA (C), the above means that $F_{A}^{1}=0$. In this case, the fraction of a certain isotopologue of acetyl-CoA with at least two labeled carbons is related to the fractions of the acetyl and CoA isotopologues, as follows:

(2) $$F_{AC}^{n}=F_{C}^{n}*F_{A}^{0}+F_{C}^{n-1}*F_{A}^{1}+F_{C}^{n-2}*F_{A}^{2}=F_{C}^{n}*F_{A}^{0}+F_{C}^{n-2}*F_{A}^{2}\;=F_{C}^{n}*\left(1-F_{A}^{1}-F_{A}^{2}\right)+F_{C}^{n-2}*F_{A}^{2}=F_{A}^{2}\left(F_{C}^{n-2}-F_{C}^{n}\right)+F_{C}^{n}$$

When we assume that acetyl is either fully labeled or unlabeled, the fraction of acetyl that is fully labeled equals the fractional contribution *FC*, and this can be calculated as:

(3) $$FC=F_{A}^{2}=\frac{F_{AC}^{n}-F_{C}^{n}}{F_{C}^{n-2}-F_{C}^{n}}$$

For every $F_{AC}^{n}$ with *n* ≥ 2, the *FC* can then be calculated. As not all of the isotopologues of acetyl-CoA and CoA were well-detected, we calculated the *FC* in each sample for all isotopologues of acetyl-CoA with *n* ≥ 2 and decided to use the one that showed the least variation within the replicates of the same cohort, which was for *n* = 8. This is demonstrated in the Supplementary Materials, datafile 1. This *FC* was used in the input for making the acetyl-CoA pie charts using TraVis Pies; for all of the other metabolites, Formula (1) was used.

### 3.5. TraVis Pies Data Visualization

This section provides a summary of how TraVis Pies operates. For more detail on how the script operates, see the Supplementary Materials, Section 3, “TraVis Pies data visualization in detail”.

The visualizations were generated in R [12], using the open source R script TraVis Pies (see code availability), relying heavily on the packages, readr [13], dplyr [14], tidyr [15] and ggplot2 [16], for data structuring and visualization. The packages extrafont [17] and forcats [18] were also used. For the web tool, the packages vroom [19], shiny [20], shinyFeedback [21] and shinyjs [22] were used. TraVis Pies operates in the following fashion.

There are several input options, all of which need a metadata input file, with sample names, a cohort column specifying the different cohorts studied and an optional normalization column. Depending on the input option chosen, the other uploaded files consist of: (a) a file containing abundance data and a file containing fractional contribution data; (b) a file containing abundance data and a file containing isotopologue data; (c) an Escher-Trace compatible input file containing abundance and isotopologue data. Working examples of each set of the input files are provided in the Supplementary Materials, data files 2, 3 and 4, and are available in the web tool. Per metabolite, a pie chart is generated for each cohort, allowing the visual comparison of abundance and labeling information between all of the cohorts for that metabolite. The radius of each circle corresponds to the average abundance of that metabolite in the specified cohort. Gray concentric circles are drawn at 25, 50, 75 and 100% of the largest average abundance for that metabolite, in order to get a visual idea of the fold change between the cohorts. If the user desires, a base 10 logarithmic scale can be used for the radius, in which case, the concentric circles are drawn at 0.1%, 1%, 10% and 100% of the largest average abundance. The fraction of the circle surface that is colored corresponds to the average amount of incorporated labels, a simplified metric for the more complex labeling patterns. The latter, known as the fractional contribution, is the ratio of nutrient-derived heavy isotopes of an element to the total amount of that element present in the metabolite [2]. The fractional contribution is also shown as a number in the center of the circle for a more detailed readout. The first cohort is taken as a reference for calculating the significances of the differences in abundance and fractional contribution for the other cohorts, which can be displayed on the graphs. In case the isotopologue data were uploaded, the user can also opt for a * to be added to the names of the cohorts for which at least one isotopologue fractions differs significantly from the reference cohort. The statistical tests are described in the Statistics and Reproducibility section.

When desired, a stand-alone high-resolution tiff figure (300 dpi) or PNG of this plot is automatically generated per metabolite, including the compound name and figure legend. For designing a pathway image, such as in this article, the user can easily enable pathway charts to produce an additional more concise image for each metabolite without a legend or compound name. This figure is more fit for being manually positioned by the user on a customized metabolic map with their preferred image editing software. The output parameters can be modified in the input section of the script, where they are described. The image sizes can be adjusted to size output figures to allow a sufficiently detailed view, depending on the number of cohorts or resolution desired.

Inkscape was used to create the metabolic map backgrounds on which we overlaid the pie plots for all of the metabolites to create Figure 1 and Figure 2, and similar figures in the Supplementary Materials.

### 3.6. Statistics and Reproducibility

As plotting additional visual indications of uncertainty on the pie charts would cause their readability to be drastically reduced, we chose to plot the significance values as text instead. For each metabolite, the first cohort is taken as a reference cohort. The significance of the difference to the reference cohort in the relative abundance and the fractional contribution is then calculated for each other cohort, as the respective *p* values, pRA and pFC. pRA is calculated by performing a *t*-test on the abundances of this cohort and the reference cohort, while pFC is calculated by performing the Kruskal–Wallis test on the fractional contributions of these cohorts. The latter is more appropriate than simple parametric models as the labeling percentage data does not show a random or Student distribution. The number of replicate samples per group are usually too small to reliably use more specific distributions fit for percentage data, such as the beta distribution. Kruskal–Wallis is also used to calculate the significance of the difference in isotopologue fractions with the reference cohort.

As can be seen in the Supplementary Materials, datafile 2, each cohort had four associated samples. These were separated into separate wells before the culturing of the cells in medium. As one out of the four samples was a control with normal glucose of ^13^C_6_-glucose in the medium, each condition had three actual replicates used for analysis.

## Data Availability

The data presented in this study are openly available and data used to generate the plots shown here are made available in the data in the Supplementary Materials and on https://github.com/vibbits/mec-shiny-apps (accessed on 23 June 2022). The TraVis Pies tool used to generate the plots shown here can be found on https://mec-shiny.vib.be/ (accessed on 23 June 2022), including instructions and documentation. The source code R script with instructions can be found on https://github.com/vibbits/mec-shiny-apps (accessed on 23 June 2022), and as a supplement to this article.

## Supplementary Materials

The following supporting information can be downloaded at: https://www.mdpi.com/article/10.3390/metabo12070593/s1, Supplementary notes, Supplementary data, TraVis Pies source code.

## Abbreviations

2DG: 2-deoxyglucose; AMA: Antimycin A; CoA: Coenzyme A; DHAP: Dihydroxyacetone phosphate; FC: Fractional contribution; GAP: D-Glyceraldehyde 3-phosphate; LC: Liquid chromatography; MS: Mass spectrometry; NAD+/NADH: Nicotinamide adenine dinucleotide (oxidized/reduced form); NT: Non-treated; pFC: *p* value for the difference of the fractional contribution; pRA: *p* value for the difference of the relative abundance; TCA: Tricarboxylic acid.
