# Supplementary material for: TraVis Pies: A Guide for Stable Isotope Metabolomics Interpretation Using an Intuitive Visualization
Source: Metabolites. 2022 Jun 25;12(7):593. doi: 10.3390/metabo12070593 (PMC9321460; doi:10.3390/metabo12070593)
Supplement: Supplementary file 1 [file metabolites-12-00593-s001.zip › Supplementary notes revised.pdf]

Supplementary notes

1. Summary

In this supplement, a roadmap for the interpretation of the TraVis Pies visualizations is provided. We first provide all the theoretical scenarios that could possibly arise when measuring the abundance and the fractional contribution, and how the combination of these metrics then further limits the possible scenarios. Second, we apply this theory to some case study compounds from the main article. Finally, we add the relations between compounds by mapping these visualizations on a biochemical map as used in the main article, consequently demonstrating how the conclusions in the main article have been obtained for pathway and metabolism level alterations.

In addition, we give a detailed rundown of the modus operandi of the TraVis Pies software.

2. Guide on interpretation of the proposed figures

2.1. Combined visualization of abundance and labeling information

2.1.1. A theoretical introduction in the interpretation of Tracer Metabolomics: possible scenario's

Key to the correct interpretation of tracer metabolomics is the necessity to simultaneously consider the abundances and fractional contribution of the target metabolites. Combining both levels in one visualization format was the aim of this publication. For the visualization, our preference went to the use of pie charts, where the radius of the pie corresponds to the relative abundance versus the reference set and the fractional contribution (see article for explanation) is indicated by the colored slice of the pie (a quarter and half corresponding to respectively 25 and 50% of the amount labeled).

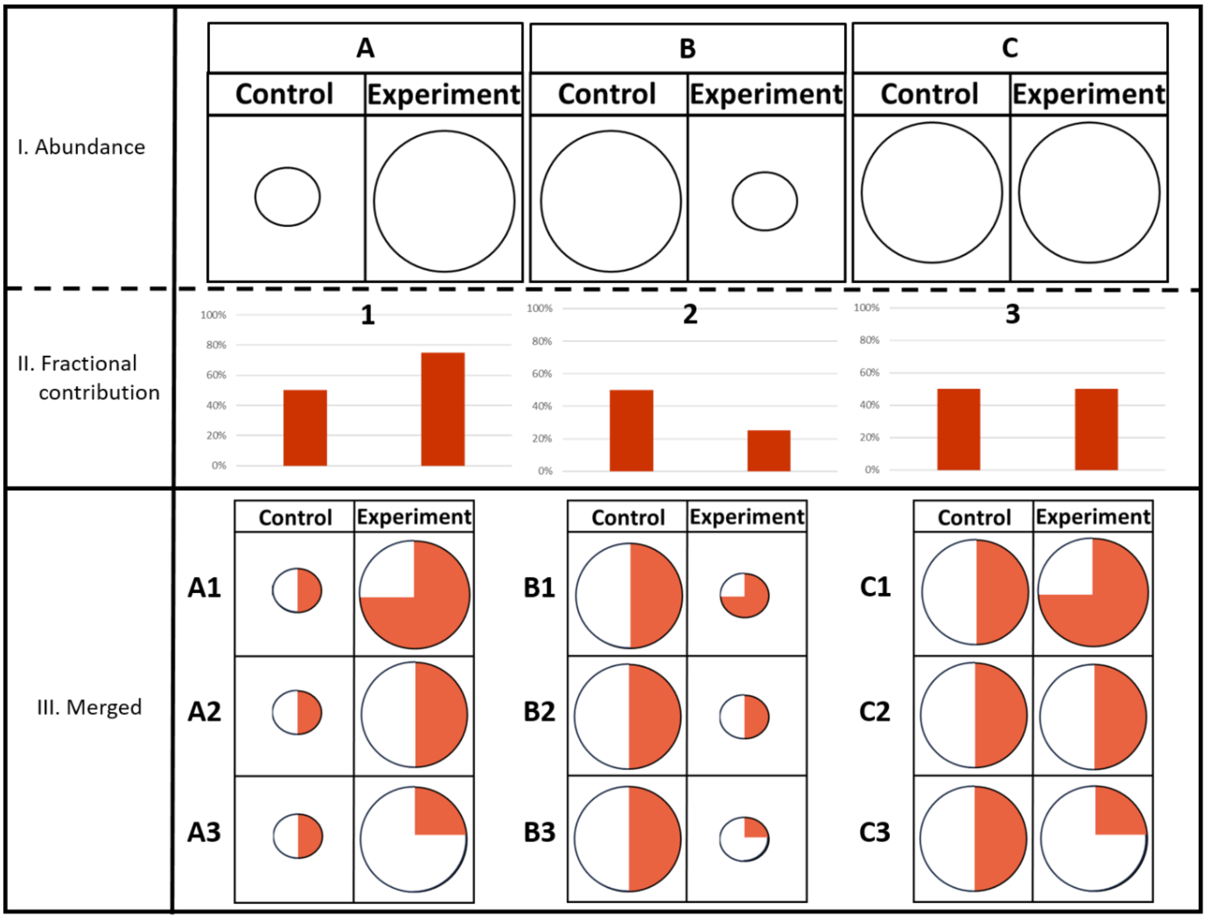

Supplementary notes Figure 1: Demonstrates principle of combining abundance and labeling information for a metabolite in one pie graph. Letters demonstrate possibly scenarios in relative abundance, number in fractional contribution, and letter-number combinations the scenari

Supplementary notes Table 1: Description of scenarios shown in Supplementary notes Figure 1

Scenario	Most Plausible explanations for experimental setup changes
A1	**Production through nutrient pathway upregulated OR** production through alternative pathways and consumption are downregulated

A2	**Consumption downregulated** or nutrient and alternative pathways upregulated in same amount relatively
A3	**Production through alternative pathways upregulated** or production through nutrient pathway and consumption are downregulated relative to production through the alternative pathways
B1	**Production through alternative pathways downregulated** or production through nutrient pathway upregulated and consumption upregulated more relatively
B2	**Consumption upregulated** or nutrient and alternative pathways downregulated in same amount relatively
B3	**Production through the nutrient pathway downregulated** or production through alternative pathways upregulated and consumption upregulated even more relatively
C1	Production through nutrient pathway upregulated and consumption upregulated **OR** production through nutrient pathway upregulated and production through alternative pathways downregulated to compensate
C2	**No changes** or nutrient and alternative pathways changed in same relative amount and consumption changed to compensate
C3	Production through alternative pathways upregulated and consumption upregulated to compensate **OR** production through alternative pathways upregulated and production through nutrient pathway downregulated to compensate

Supplementary notes Figure 1 shows the possible scenarios that can occur in a simple setup where relative levels of metabolite X from an experimental setup are compared to a control setup (control vs. treatment, healthy vs sick…). Consider that metabolite X can be produced inside the cell through multiple producing reactions, including uptake from the outside environment and multiple ways to be consumed, including excretion.

Considering solely the abundances (***part I. Abundances*** of Supplementary notes Figure 1), it is only possible to infer the changes in the relative rates of production and consumption of X. The potential scenarios linked to these changes in the relative abundance, can be caused by the following biochemical activities: in scenario A) a higher net production of X is observed in the experimental setup: this can be caused by a higher production of X through the upregulation of the producing reactions of X or by a lower consumption of X through the downregulation of the consuming reactions of X; B) a lower net production of X is observed in the experimental setup: this can be caused by a lower production of X through the downregulation of the producing reactions of X or by a higher consumption of X through the upregulation of the consuming reactions of X; C) No change is observed in the net production of X: there is no alteration, or changes in the producing and consuming reactions cancel each other out.

A better understanding of the biochemical events causing the observations in part I can be obtained by adding a labeled nutrient N that the system under study uses to produce metabolite X. The nutrient pathway results in the formation of X with labeling incorporated, while alternative pathways do not provide any label. The amount of labeling incorporated in X, summarized to one figure by the fractional contribution, gives information of the relative rate the production of X through the nutrient pathway compared to alternative pathways. The possible scenarios linked to fractional contribution, visualized in ***part II.Fractional contribution*** of Supplementary notes Figure 1, translate to the following interpretations: scenario 1) more label is incorporated in X in the experimental setup, this is caused by an upregulation of the production of X through the nutrient pathway or

because the production through alternative ‘unlabeled’ pathways is decreased; scenario 2) less label is incorporated in X in the experimental setup: opposite of 1; scenario 3) the same amount of label is incorporated in X: no relative changes in the production through nutrient and alternative pathways.

From the above, it is clear that we obtain only limited information by looking separately at the abundance (Supplementary notes Figure 1, part I) and fractional contribution (Supplementary notes Figure 1, part II). The real value of these metrics in testing the hypothesis lies in combining them. This reduces the amount of possible scenarios, often leading to one remaining explanation that is more biochemically intuitive and thus more likely. The merging of the relative abundance and fractional contribution information is visualized in ***part III.Merged*** of Supplementary notes Figure 1, while the most likely corresponding scenarios corresponding are listed in Supplementary notes Table 1.

Our pie chart visualization thus allows to quickly provide an overview of all analyzed metabolites, drawing the attention to those that display interesting changes and making a quick first hypothesis prior moving into the deeper details by examining the labeling patterns that the fractional contribution summarizes. Looking into these labeling patterns is a mandatory step in the interpretation of tracer data, because this might allow to further discern between the remaining “unsolved” scenarios proposed in the previous paragraph and allow for a deeper understanding of the metabolic changes at hand. For example, if multiple pathways connect the nutrient to M, a shift in the relative importance of one nutrient pathway to the other could noticeably change the labeling patterns in such a way that the fractional contribution barely changes. An example of this is discussed in the main article section “Verification by labeling patterns”

2.1.2. Discerning important differences between setups

So far, one downside to this format of visualization (or any other alternative with the same benefits) is that it is difficult to display the uncertainty on the relative abundance or fractional contribution without complicating the interpretation. Indeed, when measurements are relatively close to the detection limits or when they display large variations due to technical or biological reasons, the

relative abundance within a setup can vary a lot. A two or threefold difference between setups in abundance can still be due to chance under those conditions. In order to provide the ability to surmise quickly whether the difference in relative abundance between two setups is significant, the p-value calculated from a two-sided t-test comparing the relative abundances of each setup to the reference setup is added to the figure. Likewise, for the fractional contribution a p-value based on a Kruskal-Wallis test is implemented, which is more appropriate than simple parametric models as the labeling percentage data does not show a random or student distribution. The number of replicate samples per group are usually too small to reliably use more specific distributions fit for percentage data such as the beta distribution. Note that the authors do not advocate rejecting or accepting differences for individual metabolites based on their p-value alone without any further consideration of whether the observed difference makes sense or not in light of results of related metabolites.

2.1.3. Application to case study

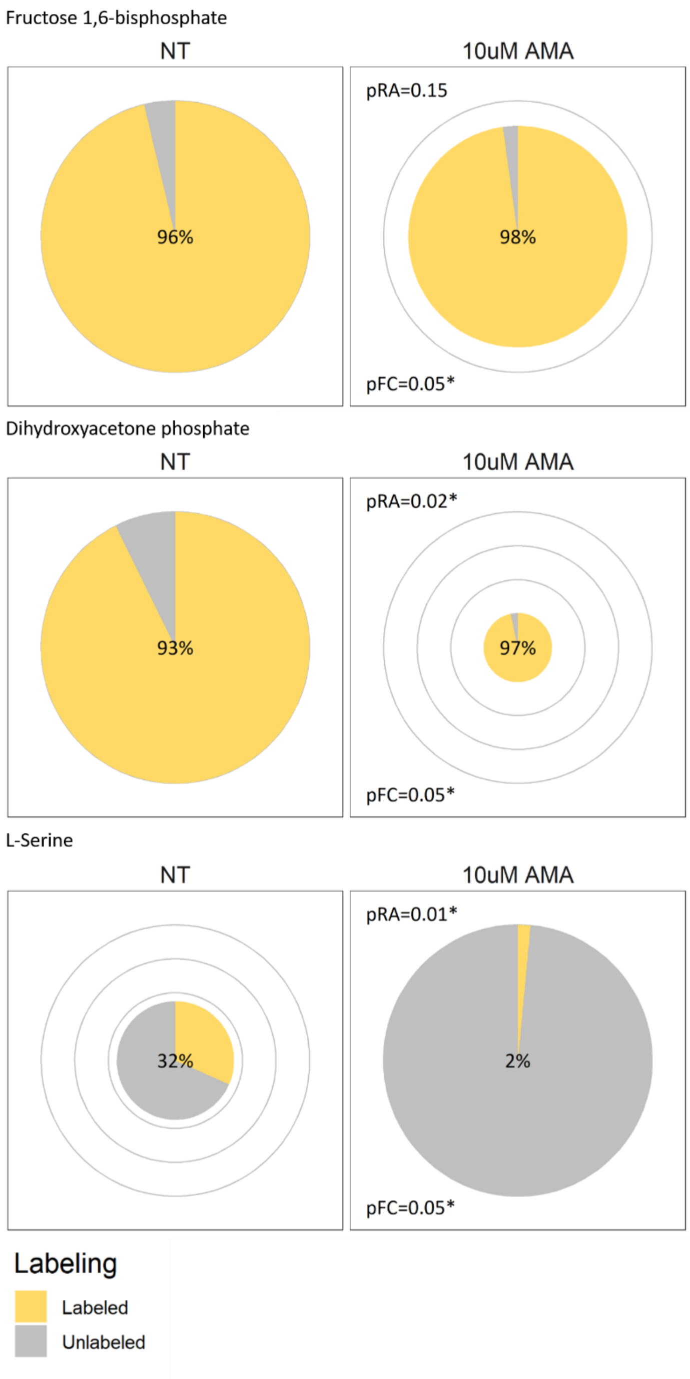

*Supplementary notes Figure 2: Examples of pie chart visualizations for several compounds of the main article, to demonstrate rules applied to non-treated (NT) or 10uM antimycin A treated (AMA). pRA and pFC indicate the significance of the difference in respectively the relative abundance or fractional contribution with the NT setup. * indicates a p value ≤0.05*

Applying the above concepts to three selected compounds from our study (here non-treated versus AMA treated 4T1 murine cancer cells) that are linked to glycolysis (Supplementary notes Figure 2).

Fructose 1,6-bisphosphate is a glycolytic intermediate. In this case, it has a similar abundance in both treated and untreated cells, and although labeling differs significantly according to the Kruskal-Wallis test, it can be considered as a less biochemically important difference. Thus, Fructose 1,6-bisphosphate does not seem to differ between untreated cells and cells treated with 10µM AMA, implying that the rates of uptake and consumption either did not change or changed in more or less the same proportion.

Dihydroxyacetone phosphate (DHAP) is converted from Fructose 1,6-bisphosphate and it is much less abundant in AMA treated cells, while the difference in labeling is only incremental. This implies that the contributions of different (unlabeled versus labeled) sources

of DHAP did not change or changed in more or less the same proportion, but the consumption did increase or -less likely- total production from all sources decreased.

Serine, an amino acid, can either be converted from glucose via 3-phosphoglycerate, or from glycine, or taken up from the medium. In AMA treated cells, it is more abundant and virtually unlabeled. From this figure it seems that while more serine is present (through production from glycine or via uptake from the medium), a strong reduction of the synthesis of serine via 3-phosphoglycerate is observed.

In all of these examples, we did not consider labeling patterns for sake of simplicity.

2.2. Visualisation on pathway

2.2.1. Concept

The next layer of information is where the pie chart figures become very useful. In the above example, we assumed that the glycolytic precursor for the discussed metabolites was not affected by treatment of the cells with AMA. In reality they are and this can change the interpretation. A difference in labeling/abundance of one metabolite might actually have been caused by a change in metabolism that happened 'upstream' rather than in the steps producing or consuming an individual metabolite. A table or list of figures such as Supplementary notes Figure 2 lacks the holistic information on which metabolites are potential precursors to other metabolites, and requires the researcher to know all relevant metabolic pathways by heart or constantly reference an external source of this information. This issue can be resolved by drawing the above visualization for multiple metabolites on a metabolic map containing relevant metabolic pathways.

Plotting the tracer data onto a metabolic map, we can now follow the labeling from the nutrient into different pathways, and quickly identify which metabolites display changes in abundance and/or fractional contribution patterns. Consequently, we can then get an idea of what is happening with these metabolites, prior to looking further into the labeling patterns at these steps.

2.2.2. Application to case study

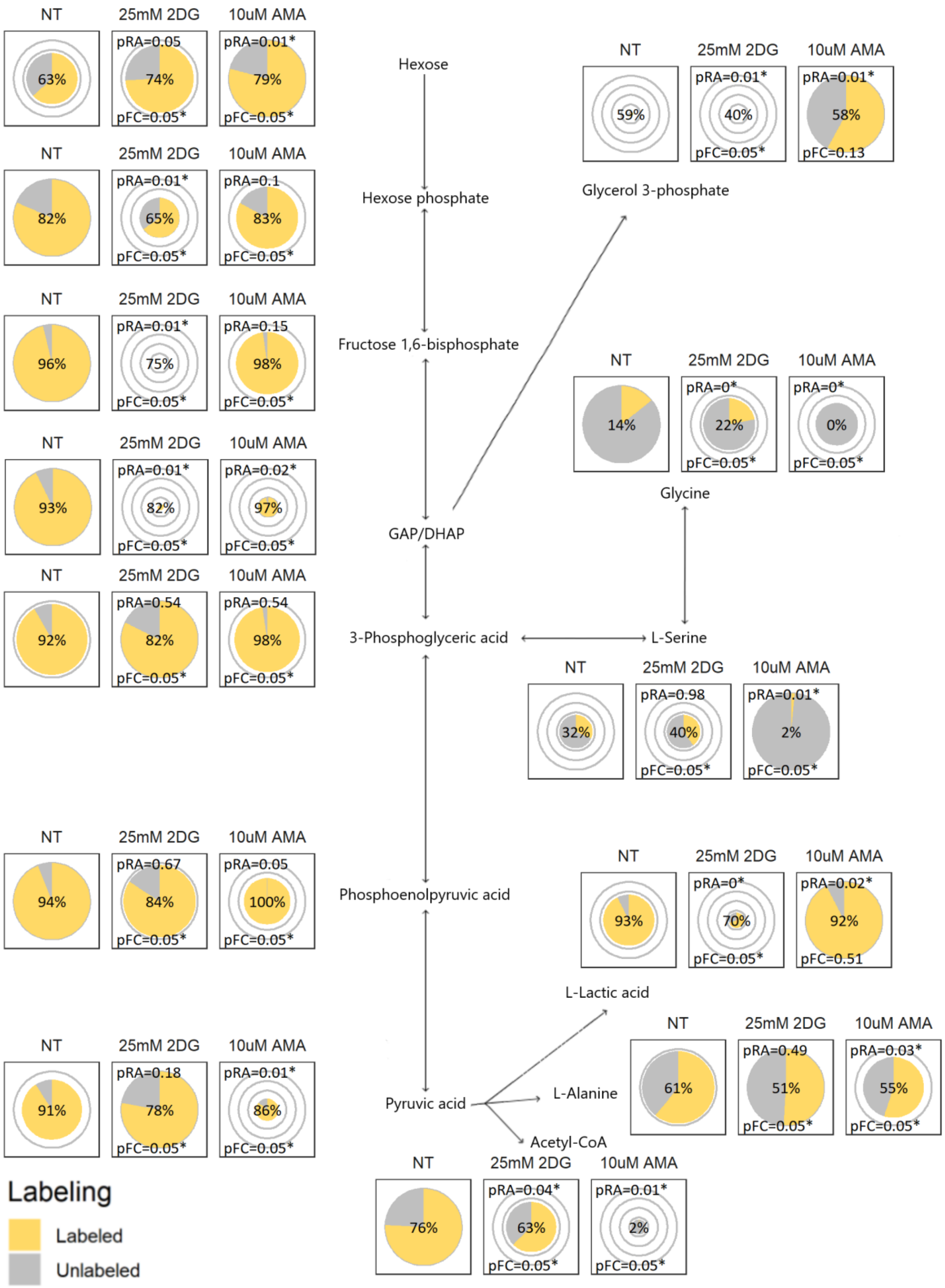

Supplementary notes Figure 3: Proposed pie chart visualization plotted on a metabolic map of the metabolization of 13C6-labelled glucose into glycolysis applied to 3 setups: non-treated (NT), 25mM 2-deoxyglucose treated (2DG) or 10uM

*antimycin A treated (AMA). pRA and pFC indicate the significance of the difference in respectively the relative abundance or fractional contribution with the NT setup. * indicates a p value ≤0.05. GAP/DHAP: figure for either D-Glyceraldehyde 3-phosphate or Dihydroxyacetone phosphate, that are in equilibrium.*

Supplementary notes Figure 3 above is the same as Figure 1 from the main article. It is an *in vitro* tracer metabolomics experiment comparing $^{13}C_6$-glucose metabolization in 4T1 murine breast cancer cells that were either: non-treated (NT), 25mM 2-deoxyglucose treated (2DG) or 10uM antimycin A treated (AMA) for 24 hours. In order to correctly and intuitively interpret the figure, we move from the nutrient down into the pathways that contain labeling in at least one of the setups. First, we try to observe the most obvious differences, then we try to link related observations together and also consider smaller differences in related metabolites to make a plausible hypothesis for what happens in subsections of the pathway. We use those partial hypotheses to generate a high-level summary linking all observations. The latter we can then use to build a hypothesis on what metabolic differences there are between setups.

Comparing NT to 2DG

- Observe the most obvious differences following nutrient through pathways of interest
    1. Hexoses are less labeled and slightly less abundant in NT than in 2DG, meaning less labeled glucose is present in NT extracts;
    2. Unlike hexoses, hexose phosphates are less abundant in 2DG conditions, and Fructose 1,6-bisphosphate and GAP/DHAP are much less abundant;
    3. Abundances 'recover' starting at 3-phosphoglyceric acid;
    4. 2DG cells have is much less lactic acid and slightly less acetyl-CoA;
    5. Unlike hexoses, hexose phosphate is less labeled, and this difference is carried through glycolysis up to pyruvic acid and its derived metabolites;
    6. More labeling appears in serine and glycine.
- Try to build a comprehensive story for observations above, we added the observations numbers from which a hypothesis follows:

- Observations 1, 2 and 4: Glycolysis is being partially blocked, as metabolites that are expected to be inhibited (hexose phosphate and fructose 1,6-bisphosphate) are indeed (much) reduced in terms of abundances, and the output of the glycolysis pathway, lactic acid, is as well;
- Observation 3 and 5: abundances are restored at 3-phosphoglyceric acid, and labeling is not reduced compared to metabolites upstream, so glucose is likely still channeled to 3-phosphoglyceric acid either by complete consumption of intermediate metabolites or by a bypass of the block;
- Observation 3 and 6: Serine and glycine seem to be more labeled and similarly abundant, and thus generated more from 3-phosphoglyceric acid in 2DG cells.

Comparing NT to AMA

- First: observe the most obvious differences following nutrient through pathways
    1. Hexoses are less labeled and slightly less abundant in NT than in AMA, meaning less labeled glucose is present in NT extracts;
    2. Hexose phosphate and fructose 1,6-bisphosphate are similarly abundant and labeled
    3. GAP/DHAP is much less abundant;
    4. Abundances are similar again at 3-phosphoglyceric acid, but pyruvic acid abundance is again reduced in AMA cells;
    5. Lactic acid is much more abundant in AMA cells, alanine is similar and acetyl-CoA is much less abundant and virtually unlabeled;
    6. Glycerol3P is much more abundant in AMA cells;
    7. In AMA cells, serine and glycine are virtually unlabeled, and serine is much more abundant while glycine is actually less abundant.
- Try to build a comprehensive story for observations above, we added the observations numbers from which a hypothesis follows:

- Observations 1, 2, 3, 4 and 6: while most early glycolytic metabolite levels are being maintained, DHAP is being consumed to generate glycerol 3-phosphate, which might indicate that the glycerol 3-phosphate shunt is activated in order to restore the intracellular NAD:NADH ratio;
- Observation 4, 5 and 7: the later glycolytic metabolites seem to be channeled to lactic acid instead of other products of glycolysis, which can also restore the intracellular NAD:NADH ratio;
- Observation 5: virtually no glucose carbons are entering the TCA cycle via acetyl-CoA.

3. TraVis Pies data visualization in detail

3.1. Introduction

This section is intended to provide technical details on the functioning of the TraVis Pies application. For a practical user guide, please see the manual provided in the TraVis pies web application or with the TraVis pies code in the supplementary material of this article.

3.2. Dependencies

Visualizations were generated in R [1], using the open source R script TraVis Pies (see code availability), relying heavily on the packages readr [2], dplyr[3], tidyr [4] and ggplot2 [5] for data structuring and visualization. The packages extrafont [6] and forcats [7] are also used. For the web tool, packages vroom[8], shiny[9], shinyFeedback[10] and shinyjs[11] and were used.

3.3. Raw input data

There are several input options, all need a metadata input file, with sample names, optionally but likely a cohort column specifying the different cohorts studied and an optional normalization column. Depending on the input option chosen, the other uploaded files consist of a) a file containing abundance data and a file containing fractional contribution data; b) a file containing abundance data and a file containing isotopologue data; c) an Escher-Trace compatible input file containing Abundance and isotopologue data.

3.4. Input module

The user can choose to either create a new standardized TraVis Pies input file from the 3 input files described in the previous section, or upload a previously created standardized TraVis Pies input file to move on faster.

Creating a new file will allow the user to upload the three input files and specify which columns contain sample names, cohorts and normalization, and which compounds should be included in the analysis. The standardized TraVis Pies input file created this way can be saved for uploading next time the app is used for this data, and will be used as input for this session.

Uploading a previously created file will perform some checks to make sure the format if the input is correct to avoid errors down the line, if this file was not manually modified no problems should be encountered. This input will then be used in the visualization module.

3.5. Input cleaner module

Read in and clean the 3 input .csv's as tibbles, for easier manipulation to ggplot2 compatible format for creating figures later on

- Clean uploaded metadata, abundance and fractional contribution or isotopologue data
  - Remove empty columns
  - Give error if no sample column present in any file
  - Give error if metadata contain samples not in the abundance or fractional contribution data
  - Fractional contribution/isotopologues: take input whether it is as fractions or percentages, convert latter to fractions
  - Allow visual inspection of the cleaned uploaded datasets as an interactive table.
- Metadata formatting
  - For inputs using 3 files (with either FC or isotopologue data) the column name that appears in all input files is taken as a potential sample name column. Lack of any such

column results in an error message. For 2- file input with Escher-Trace like format the column names starting at the third column are assumed to be the samples.

- Allow selection of normalization column from all columns not containing sample names. Add dummy normalization column containing only 1's if no normalization column present or selected.
- Allow selection of cohort column from all columns not containing sample names or normalization factor. Add a dummy cohort column with only one cohort if no cohort column is present or selected
- Other columns are dropped

Samples not present in the metadata file will be removed, in addition to compounds only present in the fractional contribution file or isotopologue file. Compounds only present in the abundance file will be considered 100% unlabeled. Generates warnings about this if appropriate.

User can choose any combination of all remaining compounds to analyze, by default all will be analyzed.

Merge the three input data sets and standardize the result to a single TraVis input tibble.

- Rename the sample column to Sample
- Set fractional contribution and, if provided, isotopologue fractions of compounds only in abundance table to 100%
- FC's below 0 (due to natural abundance correction on undetectable signals) to 0 by adding to values below 0 the smallest amount per metabolite that brings all values below 0 to 0 or above. This avoids graphical glitches in the output.
- If provided, isotopologues fractions below 0 indicate no zero correction was done before, which likely means their negative value was added to the parent isotopologue. For isotopologue fractions below 0, their absolute value is subtracted from the parent isotopologue, and their value is set to 0

- The isotopologue data is summarized to a string for each metabolite in each sample containing the fraction of each isotopologue from light to heave separated by the character "|"
- Join the abundance and fractional contribution data and, if provided, isotopologue strings with the metadata in one table, by having two entries per sample, one for its abundance and one for its fractional contribution, or three when isotopologue strings were calculated. This format is compatible with the calculations performed further on.
- If normalization requested normalized abundances are calculated and added for each sample as new rows by dividing abundances of all compounds in each sample by its normalization value

When a merged table is generated, allow any of the following.

- to view it as an interactive table
- to save the resulting standardized TraVis input file for quicker input by uploading it directly in the input module next time
- Close the input cleaner interface and continue to the visualisation interface

3.6. Visualisation module

This module shows the pie chart visualization for a compound of choice from the last dataset uploaded or created in the input module, combined with the current visualization settings. It allows users to adapt these settings, see the resulting pie chart plot for a compound of choice before the more time-consuming step of generating and downloading similar pie chart plots for all desired compounds. It also shows a potential caption for the file based on the settings chosen, which will be outputted with the figures in the output module. Below is a rundown of the settings that can be changed.

3.6.1. Compound, cohort variable, cohort order and normalization

- Compound: select compound from the dataset should be shown as an example in the visualization app
- Cohort variable: select variable that should be used to group the samples in different cohorts

- Cohorts: Select the cohorts to be shown and their order. The first cohort will be used as a reference for statistical tests
- Use normalized abundances: if available and checked, uses normalized abundances instead of raw abundances for the pie radius and statistical tests.
- Show * in cohort name if significant isotopologue difference: if available and checked, a * will be added to the cohort name for a metabolite if that cohort has any significant differences in individual isotopologue fractions with the first cohort (p <0.05) based on a Kruskal-Wallis test.

3.6.2. Chart layout options

- Maximum charts per row: The number of columns in the facet plot, equaling the maximal amount of pie charts that will be shown on one horizontal line in each figure. If the amount of cohorts exceeds this value, the next line will be used until all cohorts are plotted.
- Position FC label: Position of the label displaying the fractional contribution. If "center", the FC is shown in the center of the graph. If "slice", both the contribution of the labelled and unlabeled fractions are shown in their specific slices.
- Slice label distance from center: this is the minimal distance from pie center at which a label can be plotted if Position FC label is "slice". 1 is the maximal radius a pie can have. Normally a label appears at half the radius of the pie, but if this is smaller than minLabDist it will be plotted at minLabDist distance instead.
- FC label decimals: the amount of decimals shown in the fractional contribution labels
- Add '%' to FC label: if checked adds % to the fractional contribution label (eg. 13% instead of just 13)
- show_P: if checked hows text relating to P values of statistical significant tests on pie charts
- Use base 10 logarithmic scale for abundance: if checked, the abundance axis (i.e. the pie radius) will be on logarithmic scale. An x-fold difference in pie radius is then a 10x fold difference in abundance (i.e. a cohort with twice the reference pie radius has a 100 fold

higher abundance). Might be useful to show both pies when even when abundance differences are large, but less intuitive to interpret.

- Add legend: if checked include the labelling color legend for slice colors on the detailed figure. This is never plotted on the concise figure for pathways.
- Add compound name as title: if checked include the compound name on the detailed figure. This is never plotted on the concise figure for pathways.
- Pick colour (un)labeled fraction: select a colour to be used for the (un)labeled fraction slice of the pie
- Pick circle line colour: select a colour to be used for the concentric circles
- Set opacity: change the opacity of the pie slices to see the concentric circle lines more or less distinctly
- Circle linetypes: change the line type (none, solid, dotted…) of the concentric circles

3.6.3. Chart font options

- Select font: specify the font to be used on the result figures. To get the option to use any but the default font in a local version of this application, a separate script must be run to allow R to use the fonts stored on the machine.
- Fontsize: change fontsize of specified text on the figure

3.6.4. Generating Pie chart from input and settings

In the visualisation module, a pie chart is generated from the input data of the selected compound using the settings and the logic below

- Create a compound tibble with data of the selected compounds per sample for further processing
    - Keep metadata, fractional contribution, and depending on setting regular or normalized abundances
    - Keep only selected cohorts

    - Make the cohort variable a factor for visualisation and significance tests, and set the order of the data to match that specified by the user (1st cohort will be used as a reference)
- Summarize the data per cohort as means, add statistical test information and labels to use in the plot
    - Calculate significance (P value) of differences between cohort abundances using a two-sided t-test, and between cohort fractional contributions using a Kruskal-Wallis test.
    - Calculate means of abundance and fractional contribution per cohort
    - Per cohort, join mean and P values to one table
    - Express mean abundances relative to the mean largest abundance so all are a fraction between 0 and 1
    - Calculate the labeled fraction and the unlabeled fraction of these normalized mean abundances by multiplying them with the fractional contribution and 1-fractional contribution of the same cohort respectively
    - Regroup the data to have one entry per pie slice, meaning one labeled and one unlabeled entry for every cohort.
    - Add labels for the fractional contribution to the tibble
        - Format depending on input
        - If slice positioning requested, distance of label from center based on the normalized mean abundance (minimal distance depending on setting) and radial position of label calculated based on labeled and unlabeled fractions per cohort
    - Add labels for the P values to the tibble
- Generate the pie chart based on the cohort tibble, with details depending on settings
    - Use the tibble to generate a stacked bar chart per cohort

  - Add gridlines that will become concentric reference circles later
  - Add FC and if desired P labels at requested positions
  - Add compound names as a chart title if desired
  - Transform the stacked bar charts into pie charts by transforming to the polar coordinate system
  - Plot the data of each cohort separately in a facet grid
  - Apply final formatting to the pie charts to improve readability
- Plot this pie chart as visual feedback of the effect of the chosen settings
- The user can click the button “Save plots with these settings” if the settings are ok.

3.7. Output module

Accessed once the user clicked the button “Save plots with these settings”, this module has a few settings regarding to which figures will be generated and the plot type

- The user can select whether they want to output one or both of the following plot types
  - A detailed figure according to settings chosen
  - A figure in more concise format without compound name and legend regardless of settings, more fit for overlaying on pathways.
- The user can select whether they want to output as .png or .tiff. They have similar quality but are suited for different purposes
  - .png is more suited for screen and web display as the files are smaller
  - .TIFF uses a colour scheme that is more compatible with conventional printing, however the files are very big
- The use can make any selection from the compounds in the input to output figures for, by default all

The user can then obtain the images for the desired compounds by

- in the standalone local TraVis Pies version: selecting or copypasting a folder path to output the figures to, then clicking generate figures
- If available, in the web version: clicking on the generate figures button, then downloading the resulting zipped file when finished.

The user can then obtain the images for the desired compounds by selecting or copy-pasting a folder path to output the figures to, then clicking generate figures. A progress bar will pop up to show how many compounds still need to be processed, as this can take some time when 10’s of compounds are supplied. A .txt file containing the caption adapted to the settings chosen is also added to serve as a basis for a caption/methods section for documents including these figures.

4. Comparison of different visualization approaches

A basic version of the different types of plot discussed in section 2.1 of the article is shown here on a metabolic map, for visual comparison. Our rationale for preferring the pie charts is explained in the article.

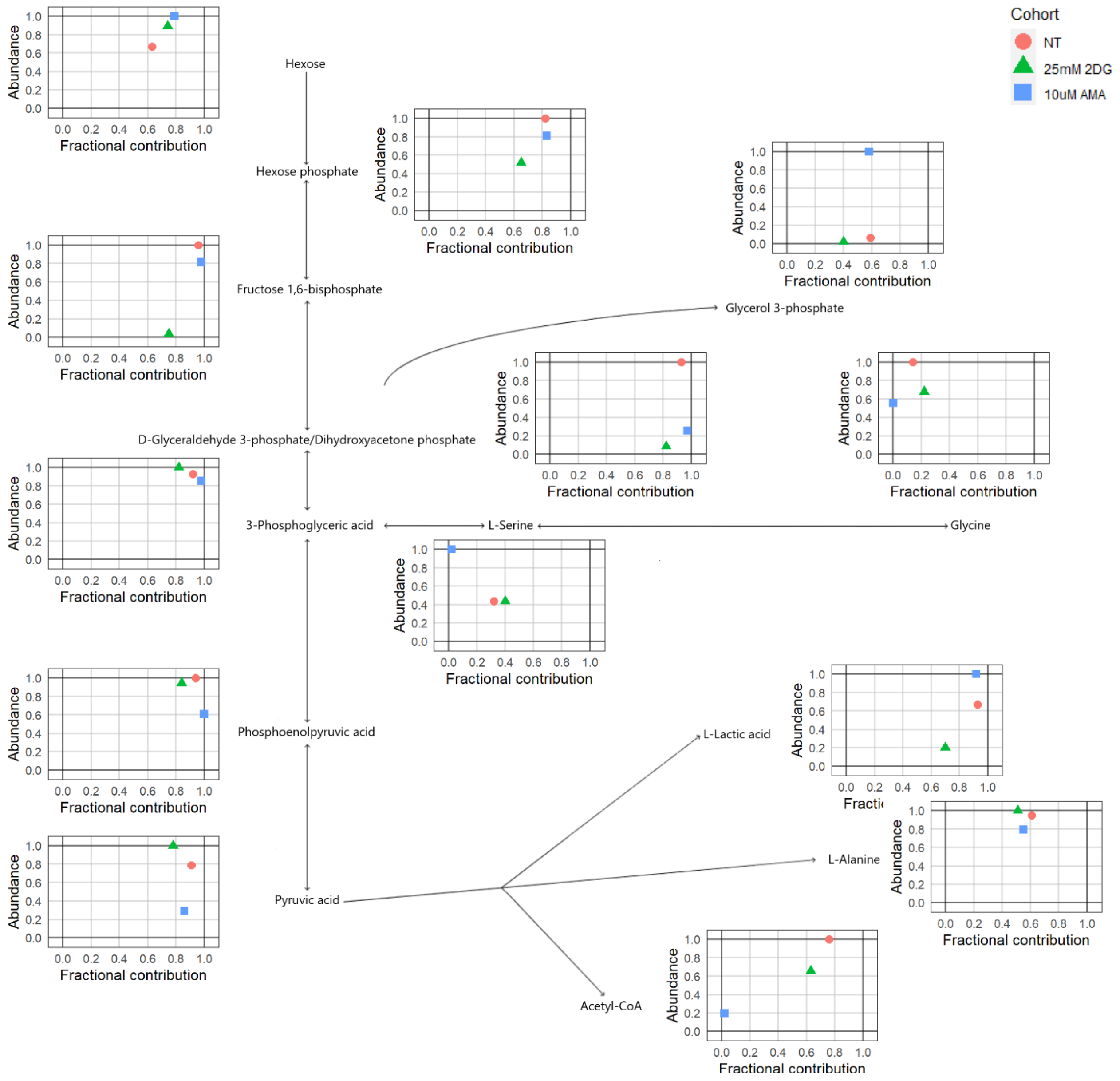

Cohort
NT
25mM 2DG
10uM AMA
Hexose
Hexose phosphate
Fructose 1,6-bisphosphate
Glycerol 3-phosphate
D-Glyceraldehyde 3-phosphate/Dihydroxyacetone phosphate
3-Phosphoglyceric acid
L-Serine
Glycine
Phosphoenolpyruvic acid
L-Lactic acid
L-Alanine
Pyruvic acid
Acetyl-CoA
Abundance
Fractional contribution

1

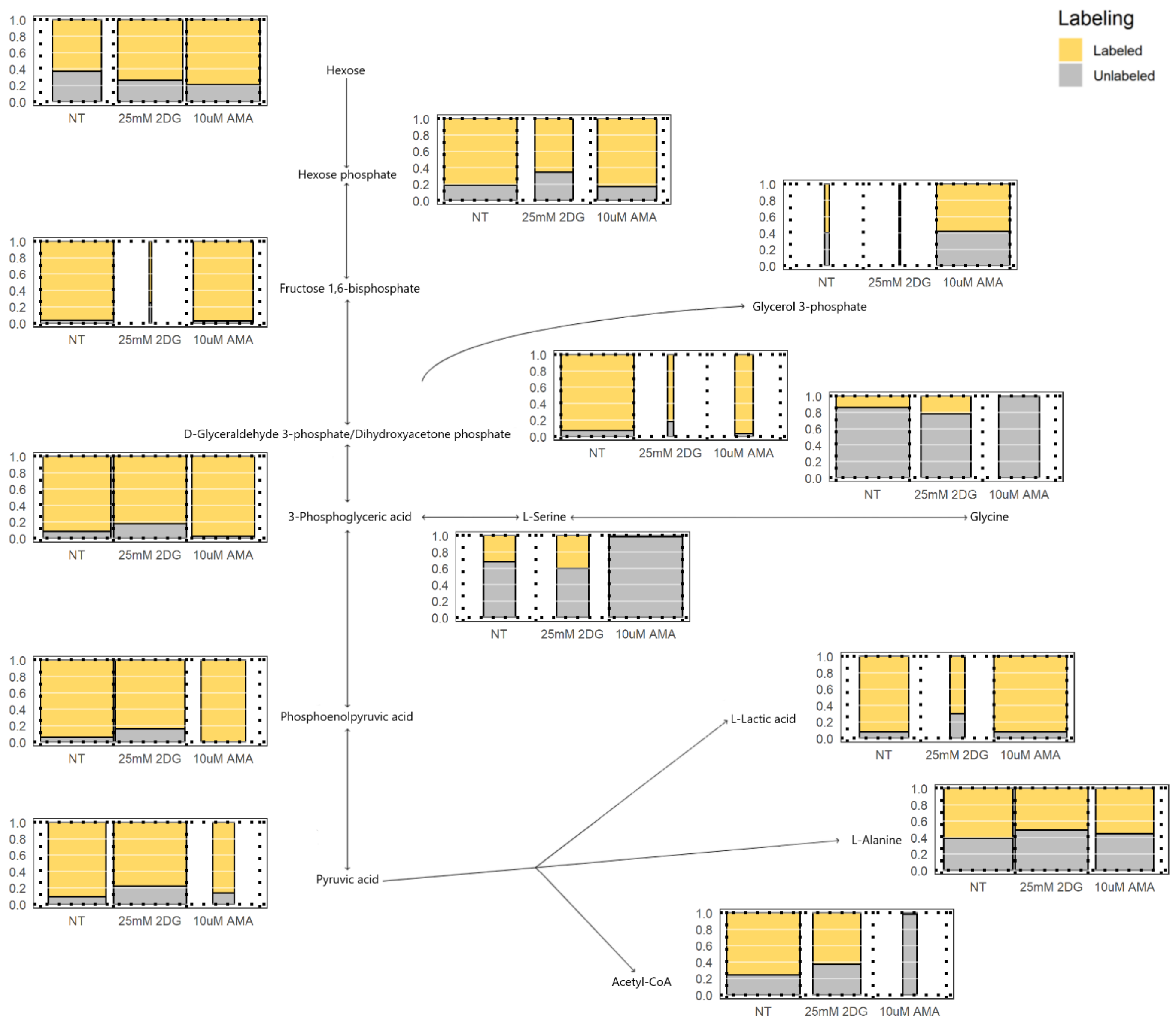
Labeling
Labeled
Unlabeled
Hexose
Hexose phosphate
Fructose 1,6-bisphosphate
Glycerol 3-phosphate
D-Glyceraldehyde 3-phosphate/Dihydroxyacetone phosphate
3-Phosphoglyceric acid
L-Serine
Glycine
Phosphoenolpyruvic acid
L-Lactic acid
L-Alanine
Pyruvic acid
Acetyl-CoA
1.0
0.8
0.6
0.4
0.2
0.0
NT
25mM 2DG
10uM AMA

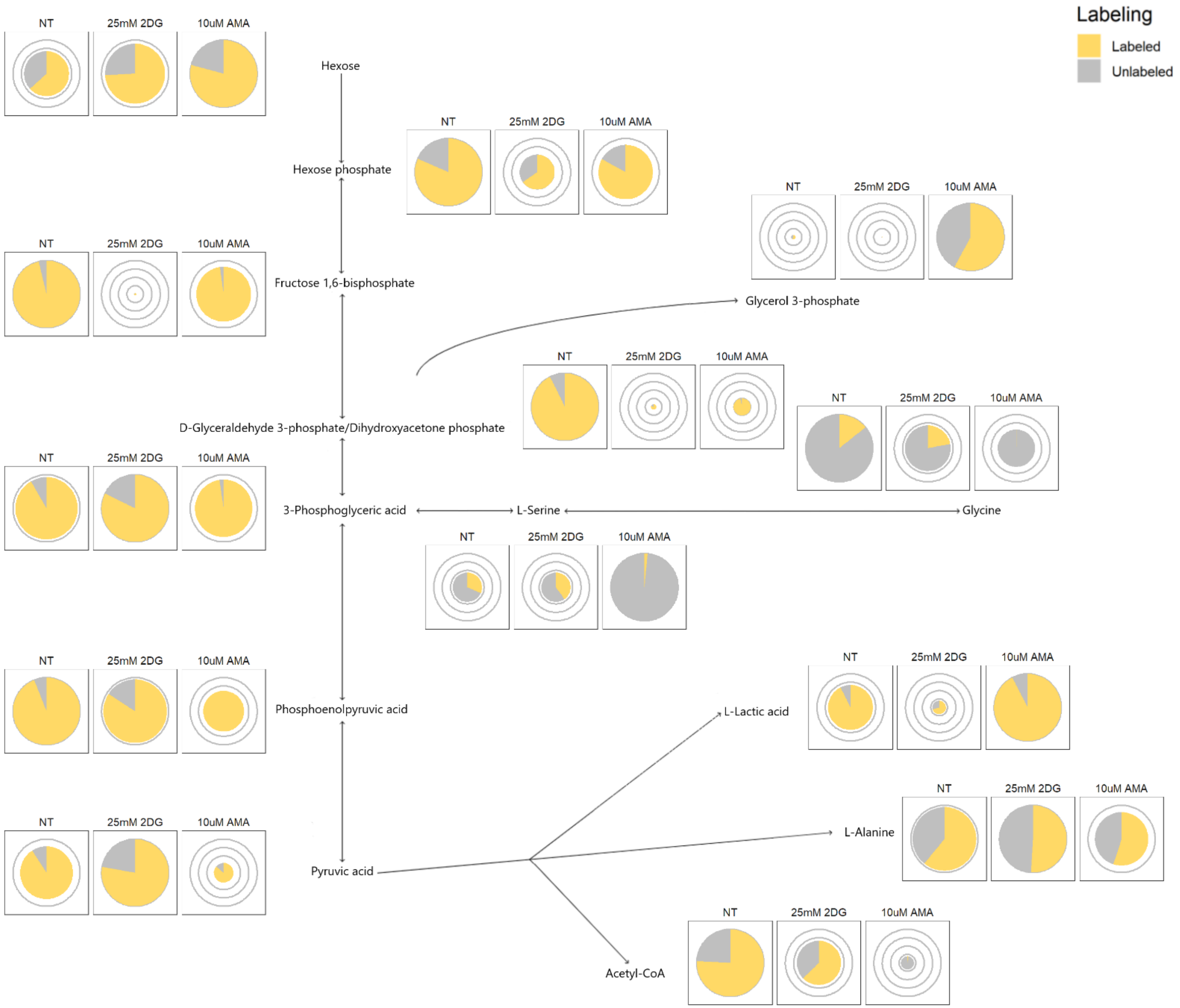

Labeling
Labeled
Unlabeled
Hexose
Hexose phosphate
Fructose 1,6-bisphosphate
Glycerol 3-phosphate
D-Glyceraldehyde 3-phosphate/Dihydroxyacetone phosphate
3-Phosphoglyceric acid
L-Serine
Glycine
Phosphoenolpyruvic acid
L-Lactic acid
L-Alanine
Pyruvic acid
Acetyl-CoA
NT
25mM 2DG
10uM AMA

5. Supplementary References

1. R Core Team. R: A language and environment for statistical computing. (2021).
2. Wickham, H. & Hester, J. readr: Read Rectangular Text Data. (2021).
3. Wickham, H., François, R., Henry, L. & Müller, K. dplyr: A Grammar of Data Manipulation. (2021).
4. Wickham, H. tidyr: Tidy Messy Data. (2021).
5. Wickham, H. ggplot2: Elegant Graphics for Data Analysis. (2016).
6. Winston, C. extrafont: Tools for using fonts. (2014).
7. Wickham, H. forcats: Tools for Working with Categorical Variables (Factors). (2021).
8. Hester, L. & Wickham, H. vroom: Read and Write Rectangular Text Data Quickly. (2021).
9. Chang, W. *et al.* shiny: Web Application Framework for R. (2021).
10. Merlino, A. & Howard, P. shinyFeedback: Display User Feedback in Shiny Apps. (2021).
11. Attali, D. shinyjs: Easily Improve the User Experience of Your Shiny Apps in Seconds. (2021).
