# Supplementary material for: TraVis Pies: A Guide for Stable Isotope Metabolomics Interpretation Using an Intuitive Visualization
Source: Metabolites. 2022 Jun 25;12(7):593. doi: 10.3390/metabo12070593 (PMC9321460; doi:10.3390/metabo12070593)
Supplement: Supplementary file 1 [file metabolites-12-00593-s001.zip › TraVis_Pies-v1.2/TraVis_Pies_manual.pdf]

Travis Pies manual and documentation

By Sam De Craemer

1. Table of contents

Contents

1. Table of contents ... 1

2. Using the TraVis pies interface ... 2

2.1. Obtaining curated tracer data ... 2

2.2. Prepare input data ... 2

2.2.1. Three .csv file input with FC ... 2

2.2.2. Three .csv file input with isotopologues ... 4

2.2.3. Two .csv file aka Escher-Trace input format ... 5

2.3. Select input method and files ... 6

2.4. Input standardizer ... 6

2.5. Visualisation ... 7

2.5.1. Note on using multiple selections for cohort and others ... 7

2.5.2. Compound, cohort variable, cohort order and normalization ... 7

2.5.3. Chart layout options ... 8

2.5.4. Chart font options ... 8

2.6. Output module ... 8

3. References ... 9

2. Using the TraVis pies interface

2.1. Obtaining curated tracer data

The TraVis pies app takes as input the metadata of the sample and either an abundance and fractional contribution value per compound per sample, or abundances and corrected isotopologue ratios. This means that other software needs to be used to obtain these values from raw data. To obtain abundances and corrected isotopologue ratios, 3 steps are required:

- Integrating peaks corresponding to compounds and their isotopologues from MS or LC/GC-MS data to obtain relative intensities for each.
- Calculating abundance as the sum of all isotopologues intensities
- Correcting the isotopologue intensities for the natural abundance of isotopologues to obtain the corrected isotopologue patterns

TraVis pies can calculate the fractional contribution from the corrected isotopologue patterns, although the user can also calculate this value elsewhere and provide the fractional contribution directly. See the formula at the end of this section.

In the article we used El-Maven (Elucidata) freeware for the first step, and the proprietary Polly Labeled LC-MS Workflow (Elucidata) to calculate abundances, isotopologue patterns and fractional contributions. Alternatives exist, note that the following list contains some examples and is not exhaustive, all are freeware but not tested by the author:

- Peak integration with support for stable isotope tracer experiments: AssayR
- Natural abundance correction: IsoCor, ICT, ElemCor
- Abundance and fractional contribution calculation: No publicly available freeware known to authors at the time of writing, usually by homemade scripts or by spreadsheet software.

The abundance is the sum of the (corrected or uncorrected) isotopologue intensities, the formula for calculating the fractional contribution is:

$$\mathrm{FC} = \frac{\sum_{i=0}^{n} i * m_i}{n * \sum_{i=0}^{n} m_i}$$

With FC being the fractional contribution, *n* the number of C atoms in the metabolite, *i* denoting the isotopologues, and m_i the corrected intensity of isotopologue *i* [1].

2.2. Prepare input data

For a new experiment, the input data will consist of either two or three .csv files. The former type takes as one of the inputs the Natural Isotope Corrected CSV Format that is used by the Escher-Trace application. The easiest way to obtain the correct format would be to modify the example data provided in the TraVis Pies web app (click the Example input download button), or with the source code on https://github.com/vibbits/mec-shiny-apps. What follows is a short description of these data files and images to support them.

2.2.1. Three .csv file input with FC

Metadata, abundance data and fractional contribution data data were calculated beforehand and will be added as separate files. **Note that this input type cannot be used if you wish to display information related to specific isotopologues**, e.g. whether at least one of the metabolites isotopologue's relative contribution differs significantly between groups. To do so, use one of the other input options.

- Metadata: Contains at least a column with sample names, optionally but likely a cohort column and optionally a normalization column containing a relative or absolute sample amount (tissue weight, cell protein content …). **Note: it is best not to include 12C samples at all. In any case, never include them in the same cohort as 13C samples, as their fractional contribution is naturally 0.**

Sample	Cohort	Normalisation
Sample01	NT	0.5295
Sample02	NT	0.4875
Sample03	NT	0.496
Sample05	25mM 2DG	0.419
Sample06	25mM 2DG	0.4075
Sample07	25mM 2DG	0.4385
Sample09	10uM AMA	0.354
Sample10	10uM AMA	0.2945
Sample11	10uM AMA	0.3175

- Abundance data: Contains the column with sample names that should have the same name as the copy in the metadata. All other columns are abundances of the compounds measured. Columns containing only a header (column B in the example below) will be removed automatically and can be left in.

1	Sample	01_Glycol	Phosphoe	L-Lactic_a	3-Phosph	Dihydroxy	Fru
2	Sample01		13758220	8.83E+09	47898548	5.87E+08	1.
3	Sample02		14613602	7.82E+09	53418136	5.83E+08	1.
4	Sample03		10292417	8.24E+09	39688820	7.61E+08	1.
5	Sample04		24365238	9.09E+09	77522603	5.47E+08	1.
6	Sample05		9240457	2.3E+09	37103665	38228619	58
7	Sample06		10201781	1.95E+09	44464608	45758035	49
8	Sample07		11207332	2.03E+09	45631468	51179700	46
9	Sample08		11851675	1.89E+09	45547887	57355307	50
10	Sample09		5551254	8.82E+09	26387118	1.11E+08	7.
11	Sample10		4065126	7.78E+09	28912807	90442872	7.
12	Sample11		5439468	7.1E+09	27046334	1.14E+08	[illegible]
13	Sample12		5006468	6.66E+09	21916018	1.04E+08	[illegible]

- Fractional contribution data: Contains the column with sample names that should have the same name as the copy in the metadata. All other columns are the fractional contributions of the compounds measured, although compounds that are 100% unlabeled can be left out here (automatically happens in some software) and will be processed correctly. The fractional contributions can be formatted as fractions or as percentages, but it is suggested to include at least three digits to avoid statistical artefacts (eg. 0.157 or 15.7%). Negative values due to natural abundance correction of undetectable isotopologues can be left in. Columns containing only a header (column B in the example below) will be removed automatically and can be left in.

	A	B	C	D	E	F	
1	Sample	01_Glycol	Phosphoe	L-Lactic_a	3-Phosph	Dihydroxy	Fru
2	Sample01		0.941667	0.92694	0.915673	0.927504	0.
3	Sample02		0.930717	0.925017	0.911365	0.920896	[illegible]
4	Sample03		0.947251	0.926163	0.923139	0.931559	0.
5	Sample04		-0.02024	0.004831	-0.0112	-0.00921	0.
6	Sample05		0.840053	0.731385	0.818737	0.821732	0.
7	Sample06		0.840389	0.686631	0.821908	0.817535	0.
8	Sample07		0.845859	0.684784	0.829815	0.823714	0.
9	Sample08		-0.02024	0.006601	-0.01168	-0.00848	0.
10	Sample09		1.000009	9.20E-01	0.970975	0.966225	0.
11	Sample10		0.996088	0.937661	0.976012	0.966487	0.
12	Sample11		1.000009	0.915786	0.981935	0.964553	0.
13	Sample12		-0.02024	0.004424	-0.00676	-0.00911	0.

2.2.2. Three .csv file input with isotopologues

The only difference with the last input option is that a file with isotopologue data is provided instead of a file with just fractional contribution data. Fractional contribution will be calculated by the application from the isotopologues. Important restrictions are:

- Contains the column with sample names that should have the same name as the copy in the metadata.
- for each metabolite, all isotopologues including the tracer element should be provided, even if no signal was detected
- the isotopologues per metabolite should be ordered from light to heavy, i.e. from M0 (no labelled isotope present) to Mn with n the total amount of the tracer element in the metabolite (only labelled isotopes present)
- the isotopologue name should be of the format metabolite_isolable (eg. L-Lactic_acid_M1 or L-Lactic acid_C13label-3). Important is that
    - the metabolite part is the same for all isotopologues of the same metabolite
    - there is always an underscore _ immediately following the metabolite part
    - there is no underscore in the isolable part. Apart from this, the isolable part can be anything as it will be ignored.

A	BP	BQ	BR	BS	BT	BU	BV	BW	
Sample	03_Amino	Glycine_PARENT	Glycine_C13-label-1	Glycine_C13-label-2	L-Alanine_PARENT	L-Alanine_C13-label-1	L-Alanine_C13-label-2	L-Alanine_C13-label-3	L-Seri
Sample01		87.09%	0.48%	12.43%	36.07%	0.31%	2.19%	61.42%	
Sample02		84.82%	0.19%	14.99%	38.76%	0.20%	2.26%	58.77%	
Sample03		84.74%	0.30%	14.96%	39.67%	0.16%	2.40%	57.77%	
Sample04		100.09%	-0.08%	-0.01%	100.36%	-0.33%	-0.03%	0.00%	
Sample05		79.52%	0.55%	19.93%	46.12%	1.78%	3.03%	49.08%	
Sample06		75.35%	0.37%	24.28%	46.86%	1.67%	3.26%	48.22%	
Sample07		78.14%	0.49%	21.37%	47.51%	1.68%	3.55%	47.27%	
Sample08		100.37%	-0.36%	-0.01%	100.60%	-0.58%	-0.02%	0.00%	
Sample09		99.66%	-0.24%	0.58%	44.25%	-0.65%	2.00%	54.40%	
Sample10		100.13%	-0.50%	0.37%	44.80%	-0.41%	1.26%	54.35%	

2.2.3. Two .csv file aka Escher-Trace input format

A file containing both abundance and Natural Isotope Corrected isotopologue data as used by the Escher-Trace application and a metadata file will be added as separate files. The metadata has the exact same format as for the three .csv file input, see details above.

Natural Isotope Corrected isotopologue data has a most requirements in common with the Escher-Trace application, although it some specifics differ, noted in *italic below*. In case the user expects to also generate graphs with Escher-Trace, it is best to adhere to the usually stricter requirements imposed by that software:

- The first row of the CSV must include the following headings in order: Metabolite, Fragment followed by the sample names. *For TraVis Pies, these sample names have to be the exact same as used in the metadata file rows.*
- Metabolite and Fragment entries should only use alpha-numeric characters. Non-*alpha-numeric characters are allowed by TraVis Pies but would be replaced with underscores ("_") by Escher-Trace.*
- Entries in Metabolite column indicate that data for a new fragment is being entered, thus only include these entries in rows containing your fragment abundance.
- *TraVis pies only works with whole molecules and not fragments. If including data measured from multiple fragments for the same metabolite (first column has the same metabolite name, see Escher-Trace guide), the first fragment provided will be assumed to be the entire molecule. Others will be discarded.* Simply put the whole molecule as the first fragment while keeping the others as lower entries, then the input can still be used in Escher-Trace without having to create a separate file.
- *The entry in the Fragment column directly below the Abundance entry is will not be used by TraVis Pies and is thus optional, it could even be left empty.* It is used as the identifier for the fragment as well as for graph titles in Escher-Trace.
- The first row of data for each fragment is assumed to be M0, the second M1, and so on.
- *Not used in TraVis Pies, only for Escher-Trace*: In order to have data appear next to specific escher metabolite node, use the bigg_ID of the desired metabolite node as the metabolite name.

A	B	C	D	E	
Metabolit	Fragment	Sample01	Sample02	Sample03	Sa
Phosphoe	Abundanc	13758220	14613602	10292417	[illegible]
	Phosphoe	0.06	0.07	0.05	
	Phosphoe	0	0	0	
	Phosphoe	0	0	0	
	Phosphoe	0.94	0.93	0.95	
L-Lactic_a	Abundanc	8.83E+09	7.82E+09	8.24E+09	
	L-Lactic_a	0.06	0.06	0.06	
	L-Lactic_a	0	0	0	
	L-Lactic_a	0.04	0.04	0.04	
	L-Lactic_a	0.9	0.9	0.9	
3-Phosph	Abundanc	47898548	53418136	39688820	3
	3-Phosph	0.07	0.08	0.07	
	3-Phosph	0	0	0	
	3-Phosph	0.03	0.03	0.04	
	3-Phosph	0.89	0.89	0.9	
Dihydroxy	Abundanc	5.87E+08	5.83E+08	7.61E+08	3
	Dihydroxy	0.06	0.07	0.06	
	Dihydroxy	0	0	0	
	Dihydroxy	0.04	0.04	0.04	
	Dihydroxy	0.9	0.9	0.91	
Fructose 1	Abundanc	1.43E+09	1.62E+09	1.76E+09	5
	Fructose 1	0.01	0.01	0.01	
	Fructose 1	0	0	0	

2.3. Select input method and files

The user can choose to either create a new standardized TraVis pies input file from the 2- or 3-input files described in section 2.2, or upload a previously created standardized TraVis pies input file to move on faster.

Creating a new file (see section 2.4) will allow the user to upload the required input files and specify which columns contain sample names, cohorts and normalization, and which compounds should be included in the file. The standardized TraVis pies input file created this way can be saved for uploading next time the app is used for this data, and will be used as input for this session.

Uploading a previously created file will perform some checks to make sure the format if the input is correct to avoid errors down the line, if this file was not manually modified (strongly discouraged!) no problems should be encountered. This input will then be used in the visualization module.

For simply testing the app, one can click the button “Use demo input” to utilize example data without having to upload a file.

2.4. Input standardizer

Upload the input .csv files with the data of the experiment. See section 2.2 for more details on these.

Depending on whether the input data is consistent between the files, the user will get appropriate errors or warnings, or will be able to further finetune the input by selecting the sample column, and a cohort and normalisation column if present.

The user will then be able to select the compounds that should be taken along, by default all compounds in the input.

If all settings are as desired, click the merge inputdata button to create a standardized TraVis pies input table, which can optionally be saved as a .csv file for quicker input next time it is used. The user can then select a folder and name for the .csv file using the Save to any directory button, or (quicker) copy-pasting a folder path to output the .csv to the textbox, then click the "Save to specified directory" button. **It is strongly discouraged to make manual modifications in this file** as it requires a very good understanding of the data structure, which is beyond the scope of this manual. Simply create a new file using the input standardizer if the original data is modified.

Click the "Continue with this data" button to proceed.

2.5. Visualisation

This module shows the pie chart visualization for a compound of choice from the last dataset uploaded or created in the input module, combined with the current visualization settings. It allows users to adapt these settings, see the resulting pie chart plot for a compound of choice before the more time-consuming step of generating and downloading similar pie chart plots for all desired compounds. It also shows a potential caption for the file based on the settings chosen, which will be outputted with the figures in the output module. Below is a rundown of the settings that can be changed.

Once the settings are as desired, click the "Save plots with these settings" button in order to proceed to the output module where images can be generated for all desired compounds using these settings.

2.5.1. Note on using multiple selections for cohort and others

For compounds, cohorts … multiple selection boxes are used, and these can be confusing at the start. Whether an item in the box is selected (blue) or not (gray) does not affect the output, only whether it is present in the box or not. You can delete an item, or add an item that is available but not currently present in the box by clicking in the box and selecting it from the dropdown list showing all available options currently not present in the box.

2.5.2. Compound, cohort variable, cohort order and normalization

- Compound: select compound from the dataset should be shown as an example in the visualization app
- Cohort variable: select variable that should be used to group the samples in different cohorts
- Cohorts: Select the cohorts to be shown and their order. The first cohort will be used as a reference for statistical tests
- Use normalized abundances: if available and checked, uses normalized abundances instead of raw abundances for the pie radius and statistical tests.
- Show * in cohort name if significant isotopologue difference: if available and checked, a * will be added to the cohort name for a metabolite if that cohort has any significant differences in individual isotopologue fractions with the first cohort ($p < 0.05$) based on a Kruskal-Wallis test.

2.5.3. Chart layout options

- Maximum charts per row: The number of columns in the facet plot, equaling the maximal amount of pie charts that will be shown on one horizontal line in each figure. If the amount of cohorts exceeds this value, the next line will be used until all cohorts are plotted.
- Position FC label: Position of the label displaying the fractional contribution. If “center”, the FC is shown in the center of the graph. If “slice”, both the contribution of the labelled and unlabeled fractions are shown in their specific slices.
- Slice label distance from center: this is the minimal distance from pie center at which a label can be plotted if Position FC label is “slice”. 1 is the maximal radius a pie can have. Normally a label appears at half the radius of the pie, but if this is smaller than minLabDist it will be plotted at minLabDist distance instead.
- FC label decimals: the amount of decimals shown in the fractional contribution labels
- Add ‘%’ to FC label: if checked adds % to the fractional contribution label (eg. 13% instead of just 13)
- show_P: if checked hows text relating to P values of statistical significant tests on pie charts
- Add legend: if checked include the labelling color legend for slice colors on the detailed figure. This is never plotted on the concise figure for pathways.
- Use base 10 logarithmic scale for abundance: if checked, the abundance axis (i.e. the pie radius) will be on logarithmic scale. An x-fold difference in pie radius is then a 10^x fold difference in abundance (i.e. a cohort with twice the reference pie radius has a 100 fold higher abundance). Might be useful to show both pies when even when abundance differences are large, but less intuitive to interpret.
- Add compound name as title: if checked include the compound name on the detailed figure. This is never plotted on the concise figure for pathways.
- Pick colour (un)labeled fraction: select a colour to be used for the (un)labeled fraction slice of the pie
- Pick circle line colour: select a colour to be used for the concentric circles
- Set opacity: change the opacity of the pie slices to see the concentric circle lines more or less distinctly
- Circle linetypes: change the line type (none, solid, dotted…) of the concentric circles

2.5.4. Chart font options

- Select font: specify the font to be used on the result figures. To get the option to use any but the default font in a local version of this application, a separate script must be run to allow R to use the fonts stored on the machine. Ask Sam if desired.
- Fontsize: change fontsize of specified text on the figure

2.6. Output module

Accessed once the user clicked the button “Save plots with these settings”, this module has a few settings regarding which figures will be generated and the plot type

- The user can select whether they want to output one or both of the following plot types
    - Stand-alone: A detailed figure, depending on chosen settings can contain legend and title .
    - Pathway-compatible: A figure in more concise format never with compound name and legend regardless of settings, more fit for overlaying on pathways.

- The user can select whether they want to output as .png or .tiff. They have similar quality but are suited for different purposes
    - .png is more suited for screen and web display as the files are smaller
    - .TIFF uses a colour scheme that is more compatible with conventional printing, however the files are very big
- The user can make any selection from the compounds in the input to output figures for, by default all

The user can then obtain the images for the desired compounds by selecting or copy-pasting a folder path to output the figures to, then clicking generate figures. A progress bar will pop up to show how many compounds still need to be processed, as this can take some time when 10's of compounds are supplied. A .txt file containing the caption adapted to the settings chosen is also added to serve as a basis for a caption/methods section for documents including these figures.

3. References

1. Buescher, J.M.; Antoniewicz, M.R.; Boros, L.G.; Burgess, S.C.; Brunengraber, H.; Clish, C.B.; DeBerardinis, R.J.; Feron, O.; Frezza, C.; Ghesquiere, B.; et al. A roadmap for interpreting 13C metabolite labeling patterns from cells. *Curr. Opin. Biotechnol.* **2015**, *34*, 189–201, doi:10.1016/j.copbio.2015.02.003.
