# Supplementary material for: TraVis Pies: A Guide for Stable Isotope Metabolomics Interpretation Using an Intuitive Visualization
Source: Metabolites. 2022 Jun 25;12(7):593. doi: 10.3390/metabo12070593 (PMC9321460; doi:10.3390/metabo12070593)
Supplement: Supplementary file 1 [file metabolites-12-00593-s001.zip › TraVis_Pies-v1.2/www/license.pdf]

Nucleomics Core
A VIB FACILITY

This work is owned by **the Nucleomics Core**, the 'sequencing' facility of **VIB**.

This work is licensed under a Creative Commons Attribution-ShareAlike 3.0 Unported License

You are free to: Share — to copy, distribute and transmit the work Remix — to adapt the work

Under the following conditions: Attribution — You must attribute the work referring to the original author and licensor (**VIB**) (but not in any way that suggests that they endorse you or your use of the work)

Share Alike — If you alter, transform, or build upon this work, you may distribute the resulting work only under the same or similar license to this one.

This work is licensed under a Creative Commons Attribution-ShareAlike 3.0 Unported License.
