# Supplementary figures and images for: TraVis Pies: A Guide for Stable Isotope Metabolomics Interpretation Using an Intuitive Visualization
Source: Metabolites. 2022 Jun 25;12(7):593. doi: 10.3390/metabo12070593 (PMC9321460; doi:10.3390/metabo12070593)

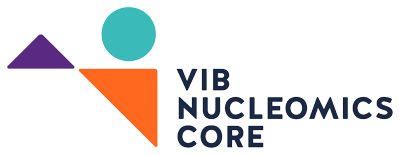

Supplement: Supplementary file 1 [file metabolites-12-00593-s001.zip › TraVis_Pies-v1.2/www/logo.png]
